# Risk factors for *Aedes aegypti* household pupal persistence in longitudinal entomological household surveys in urban and rural Kenya

**DOI:** 10.1186/s13071-020-04378-7

**Published:** 2020-10-01

**Authors:** Harun N. Ngugi, Sindiso Nyathi, Amy Krystosik, Bryson Ndenga, Joel O. Mbakaya, Peter Aswani, Peter S. Musunzaji, Lucy W. Irungu, Donal Bisanzio, Uriel Kitron, A. Desiree LaBeaud, Francis Mutuku

**Affiliations:** 1 School of Biological Sciences, Department of Zoology, University of Nairobi, Nairobi, Kenya; 2 Department of Biological Sciences, Chuka University, Chuka, Kenya; 3 Department of Epidemiology and Population Health, School of Medicine, Stanford University, Stanford, CA USA; 4 Department of Pediatrics, Division of Infectious Diseases, School of Medicine, Stanford University, Stanford, CA USA; 5 Centre for Global Health Research, Kenya Medical Research Institute, Kisumu, Kenya; 6 Vector borne Disease Unit, Msambweni Hospital, Msambweni, Kenya; 7 RTI International, Washington, DC USA; 8 Epidemiology and Public Health Division, School of Medicine, University of Nottingham, Nottingham, UK; 9 Department of Environmental Sciences, Emory University, Atlanta, GA USA; 10 Department of Environment and Health Sciences, Technical University of Mombasa, Mombasa, Kenya

**Keywords:** *Aedes aegypti*, Pupal persistence, Pupal abundance, Vector ecology, Vector surveillance, Spatial analysis, GAMMs, Dengue, Zika, Chikungunya

## Abstract

**Background:**

*Aedes aegypti* is an efficient vector of several arboviruses of public health importance, including Zika and dengue. Currently vector management is the only available avenue for disease control. Development of efficient vector control strategies requires a thorough understanding of vector ecology. In this study, we identified households that are consistently productive for *Ae. aegypti* pupae and determined the ecological and socio-demographic factors associated with the persistence and abundance of pupae in households in rural and urban Kenya.

**Methods:**

We collected socio-demographic, environmental and entomological data monthly from July 2014 to June 2018 from 80 households across four sites in Kenya. Pupae count data were collected *via* entomological surveillance of households and paired with socio-demographic and environmental data. We calculated pupal persistence within a household as the number of months of pupal presence within a year. We used spatially explicit generalized additive mixed models (GAMMs) to identify the risk factors for pupal abundance, and a logistic regression to identify the risk factors for pupal persistence in households.

**Results:**

The median number of months of pupal presence observed in households was 4 and ranged from 0 to 35 months. We identified pupal persistence in 85 house-years. The strongest risk factors for high pupal abundance were the presence of bushes or tall grass in the peri-domicile area (OR: 1.60, 95% CI: 1.13–2.28), open eaves (OR: 2.57, 95% CI: 1.33–4.95) and high habitat counts (OR: 1.42, 95% CI: 1.21–1.66). The main risk factors for pupal persistence were the presence of bushes or tall grass in the peri-domicile (OR: 4.20, 95% CI: 1.42–12.46) and high number of breeding sites (OR: 2.17, 95% CI: 1.03–4.58).

**Conclusions:**

We observed *Ae. aegypti* pupal persistence at the household level in urban and rural and in coastal and inland Kenya. High counts of potential breeding containers, vegetation in the peri-domicile area and the presence of eaves were strongly associated with increased risk of pupal persistence and abundance. Targeting households that exhibit pupal persistence alongside the risk factors for pupal abundance in vector control interventions may result in more efficient use of limited resources.
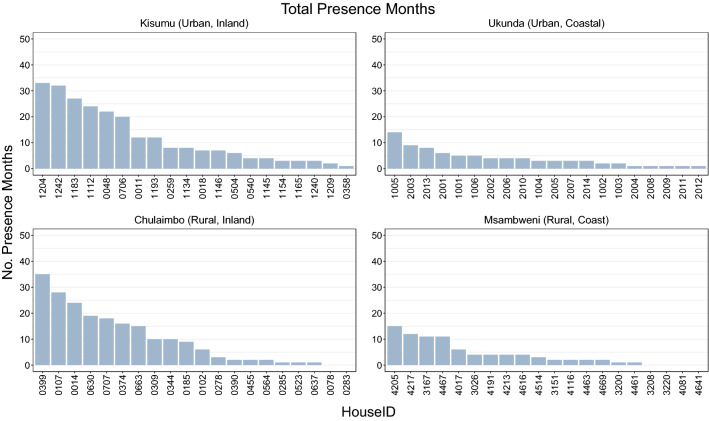

## Background

*Aedes (Stegomyia) aegypti* (Diptera: Culicidae), a mosquito well adapted to human settlements [1], is an efficient vector of several arboviruses of public health concern [2, 3] such as dengue, chikungunya and Zika viruses. Infections caused by these viruses pose a significant public health risk in tropical and sub-tropical countries. Dengue and chikungunya have been reported in Kenya recently [4–9], particularly along the coast, where prevailing climatic, environmental and socio-demographic factors favor the proliferation of vector populations [10–13].

Traditionally, determination of the risk of dengue, Zika, and chikungunya disease outbreaks in high risk regions has been based on larval surveillance and *Stegomyia* indices [14–17]. However, recent studies have shifted focus to include pupae in the surveillance of *Aedes* spp. immatures as a measure of disease outbreak risk [18–20] as pupae are a reliable proxy for adult mosquito abundance [21–24]. Lack of viable vaccines and effective therapies for dengue and other emerging arboviral infections leaves vector control as the only known disease control measure [25–27]. Given the suitability of domestic breeding habitats for *Ae. aegypti*, mosquito management within households remains central to the control of DENV and other arboviruses. As such, adequate knowledge of the factors driving abundance and persistence of *Ae. aegypti* in and around household areas is vital to the design and implementation of effective vector control strategies.

*Aedes aegypti* mosquitoes primarily breed in water-holding containers. Various factors influence infestation of containers in domestic areas with *Ae. aegypti* immatures. These include container characteristics such as location (indoors *vs* outdoors, shaded area *vs* non-shaded area), type of container (car tires, vehicle parts or drums) and size of containers [12, 20, 28, 29]. The ecology of containers such as the presence of detritus in water, water temperature and surrounding habitat (type of vegetation) increases risk of mosquito infestation [21, 30–32]. Human behavior and socio-demographic characteristics such as socio-economic status and water management methods further influence risk of infestation by *Aedes* immatures [16, 29, 30, 33–35].

The role of microclimate and larger scale human housing factors in determining container infestation has also been examined [29, 36, 37], and households surrounded by vegetation were consistently found to host *Ae. aegypti* immatures. In addition, several studies found that *Ae. aegypti* abundance exhibits spatial heterogeneity within neighborhoods and specific larval habitats within those neighborhoods produce more pupae than others [38–40]. Spatial clustering of pupae and the persistence of such clusters at the household level has also been examined [41] where abundance trends were found to be highly focal (~30 m radii) and unstable over time.

Incidence of consistently positive households (households with at least one container having *Ae. aegypti* immatures) during every inspection cycle suggests the existence of certain factors unique to such locations that warrant further investigation. Moreover, data on the household characteristics responsible for persistence of immature *Ae. aegypti* mosquitoes, particularly pupae, in Kenya are lacking. Understanding factors that drive pupal persistence in the household environment will contribute valuable information to the design of efficient and targeted vector control strategies by policy makers. In this study, we identified households that are consistently productive for *Ae. aegypti* pupae (i.e. persistent households) and determined the ecological and socio-demographic factors associated with the persistence and abundance of pupae in households in rural and urban Kenya.

## Methods

### Data collection

**Fig. 1:**
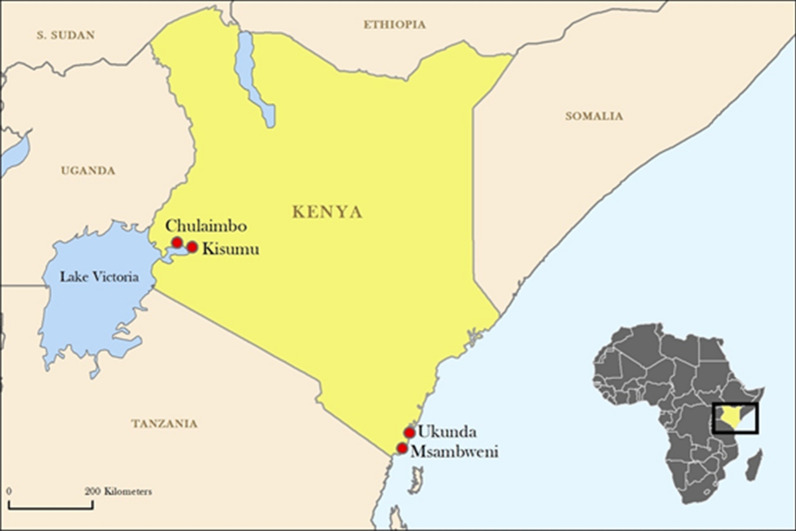
Locations of the four data collection sites in Kenya. Data were collected from 20 households per site in Chulaimbo (a rural inland site), Kisumu (an urban inland site), Msambweni (a rural coastal site) and Ukunda (an urban coastal site)

We used data collected from a total of 80 households in four sites in Kenya. In order to capture a range of possible human and environmental factors that influence vector ecology, sites were chosen based on urbanicity (rural and urban) and region (coastal and inland). Data were collected from 20 households per site in Chulaimbo (0°2′8.592″S, 34°37′15.6″E), a rural inland site, Kisumu (0°5′15.22478″S, 34°46′22.3284″E), an urban inland site, Msambweni (4°28′0.0114″S, 39°28′0.12″E), a rural coastal site and Ukunda (4°17′59.9994″S, 39°34′59.8794″E), an urban coastal site (Fig. 1). The four study sites were described in previous studies [12]. Demographic, environmental and entomological data were collected monthly from each household over a period of approximately four years beginning in July 2014 and ending in June 2018, full data description following. Climate in the regions of Kenya sampled is generally characterized as having a long-dry season (January–March), a long-rainy season (April–June), a cool-dry season (July–September) and a short-rainy season (October–December). As such, the monthly resolution and longitudinal nature of data collection allow observation of both within-year seasonal variation and overall across year variation in pupal persistence and abundance.

#### Household and demographic data

Household and demographic data collection was conducted by trained teams. Data collectors sampled 20 houses per site per month during the study period. The initial set of households included in the study within each site were chosen using random sampling of households within the study area from a 2014 census enumeration list. Each household chosen was contacted in order to obtain consent from the head of the household. If consent was obtained, the household was visited monthly throughout the duration of the study. If a sampled household did not consent to participate in the study, the most immediate neighbor was contacted. If a household that had been sampled and included in the study was unavailable for data collection at a given point in the study, the closest adjacent household was sampled in its place as a substitute. In instances where data collectors were unable to survey a given household multiple times, an attempt was made to survey the same substitute household, though this was not always possible. A household and its substitutes were treated as a single household in all analyses. Analyses were repeated with substituted time points for these houses excluded to test the sensitivity of our model to these substitutions (Additional file 1: Text S1).

During demographic surveys information was collected from households using paper forms (Additional file 1: Text S2). Variables surveyed include use of bed nets, use of mosquito repellent coils, presence of open eaves, presence of grass or bushes outside the house and number of household occupants on the night prior to sampling. Where applicable, data collectors attempted to observe the relevant variable as opposed to recording a reported response.

#### Pupae surveillance via entomological surveys

*Aedes aegypti* pupae counts were obtained from surveyed households *via* monthly entomological surveillance protocols (Additional file 1: Text S3). The entomological surveillance protocol for pupal counts is described in detail elsewhere [12]. Briefly, to estimate the total number of pupae in the household area, data collectors searched the areas inside the households and in the peri-domicile area for containers that could contain pupae, e.g. coconut husks, plastic containers, tree-holes, water troughs, jerry cans, and tires. Each container was documented (size and type) and surveyed to identify any pupae present. If any pupae were observed, the species and genus were catalogued. The number of containers per household, as well as the number of pupae per container were recorded. The total number of pupae during a household visit in a given month is used as the pupae count for the household, while the number of containers sampled is a proxy for habitat availability and the sampling effort applied.

#### Environmental data collection

Climate data was recorded hourly by hobo-loggers. Two temperature loggers (HOBO^®^ Onset data loggers, Onset Computer Corporation, 470 Bourne, MA, USA) were installed under the eaves of two houses within each of the four study areas. Daily temperature means were obtained from the land logger data and missing data were taken from logger data obtained from the paired site where possible and otherwise imputed with publicly available data from Weather Underground (www.wunderground.com; weather station codes for the coastal and western sites are HKMO and HKKI respectively) [42]. Missing data were imputed by adjusting available data from the paired site or Weather Underground by the slope and intercept of a linear regression equation based on the relationship between the two datasets. For rainfall, all measurements were taken from the National Oceanic and Atmospheric Administration (NOAA) Africa Rainfall Climatology (ARC) data at 0.1° × 0.1° spatial resolution [43]. The ARC dataset is produced using a combination of rainfall gauge measurements and METEOSAT satellite data to provide gridded rainfall estimates.

### Data analysis

#### Pupal persistence and spatial analysis

Households were defined as persistent within a year if any pupae were found in the household in at least 3 of the 12 months surveyed within that year. A cut-off of 3 consecutive/non-consecutive months was chosen based on an examination of the annual pupal presence data. Data were collected over 4 years (July 2014-June 2018). This resulted in persistence data corresponding to 320 house-years, i.e. 4 years per household for 80 households.

*Aedes aegypti* mosquitos’ average dispersal range has been found to be approximately 50 m to 100 m [44–46]. We used the Moran’s I statistic to test for spatial autocorrelation of pupae counts due to possible movement of adult *Ae. aegypti* between households [42]. This allows us to evaluate spatial correlation of pupae counts between neighboring households. The Moran’s I was conducted using household latitude and longitude values and a distance-based neighbor approach. Households were defined as neighbors if they were within a 150 m radius of one another. We tested for spatial autocorrelation amongst houses in overall pupae counts (pupae collected over the entire data collection period) as well as seasonal pupae counts (long-dry season 2014, short-rainy season 2014, etc. for a total of 16 seasons). We conducted a sensitivity analysis of this test by testing a range of radii around the house of 50 m to 250 m (Additional file 1: Text S4).

#### Identifying risk factors for pupal persistence and abundance

We used a generalized additive mixed model (GAMM) framework to model the risk factors of pupal persistence and abundance in households in Kenya [47]. GAMMs allow modeling of linear and non-linear effects using penalized regression splines. GAMMs were used to investigate the possible non-linear effect of seasons, as well as other factors such as habitat counts, temperature and rainfall. We built separate GAMMs to evaluate pupal persistence and pupal abundance. In the abundance model we used a proportional-odds model framework [48, 49]. The outcome was monthly pupae count categorized into one of four groups: zero (0 count); low (0–15 count); intermediate (15–30 count); or high (> 30 count).

We built a separate regression model to evaluate the risk factors for pupal persistence. The persistence model evaluates the risk factors for continued pupal presence in a household (> 3 months of pupal presence in a year). We used GAMMs to perform logistic regression to model the risk factors for within-year pupal persistence. We included house and site in all models as nested random effects. We accounted for spatial autocorrelation by including a spatial term modeled *via* geosplines on the latitude and longitude of the households [50–52]. For the abundance models, we specified an overall model using data from both the coastal (Msambweni and Ukunda) and inland (Chulaimbo and Kisumu) locations, and additional separate models for the coast and inland locations to account for any potential differences between the two regions. We included average habitat count, and other demographic risk factors such as number of rooms and roofing material as terms in all models. All the factors listed below were included as potential predictors in all models so as to evaluate their influence of pupal abundance and persistence. For the abundance models, we also tested models including a 1-month lag in pupal abundance term. This term was not included in the final model. The authors reasoned that any correlation in pupal abundance at the household level would be captured by the house random effect. Model fit statistics (AIC and BIC) and residual analysis were used to select which variable (temperature/rain or month) best captured environmental trends (Additional file 1: Table S1). Additional details on model building are included in the supplementary materials (Additional file 1: Text S1).

#### Statistical tools

All statistical analyses were conducted in the R programming language [53]. Moran’s I tests were conducted using the *spdep* R package [54]. Preparation and visualization of spatial data was conducted in ArcGIS (Version 10.8). We used the BayesX software package, *via* its interface R2BayesX, to build GAMMs and examine their output [52].

## Results

Demographic, environmental and entomological data were collected from 80 households in four sites (Fig.1, Table 1) from July 2014 to June 2018. Characteristics of the households included at baseline in the study are shown in Table 2. There were some differences in the household characteristics of the coast and inland households at baseline. Notably more houses in the west had mud walls compared to those at the coast. The distribution of the number of rooms per house was similar between both locations. Iron sheet roofing was more common at the coastal site (32/40 houses) than the inland site (18/40 houses). Most houses in both sites indicated bed net use (40/40 in the inland and 38/40 in the coast sites). Insecticide and mosquito repellent coil use were relatively low in both locations.

**Table 1:** Overall site characteristics. General environmental and demographic characteristics of the 4 data collection sites

Site	Households	Urban/rural	Coastal/inland	Population density (no./km^2^)	Elevation (m)	Coordinates
Kisumu	20	Urban	Inland	15,000	1100	0°5′15.22478″S, 34°46’22.3284″E
Chulaimbo	20	Rural	Inland	500	1328	0°2′8.592″S, 34°37′15.6”E
Ukunda	20	Urban	Coast	2000	8	4°17′59.9994″S, 39°34′59.8794”E
Msambweni	20	Rural	Coast	460	4	4°28′0.0114″S, 39°28′0.12″E

**Table 2:** Household characteristics at the baseline

Household characteristic	Overall (*N* = 80) *n* (%)	West (*N* = 40) *n* (%)	Coast (*N* = 40) *n* (%)
House wall material			
Mud	41 (51)	24 (60)	17 (43)
Cement	39 (49)	16 (40)	23 (58)
House roof material			
Iron sheet	50 (62)	32 (80)	18 (45)
Grass	22 (28)	1 (3)	21 (53)
Asbestos/tile	8 (10)	7 (18)	1 (3)
No. of rooms			
< 3	24 (30)	12 (30)	12 (30)
3–4	33 (41)	16 (40)	17 (43)
≥ 5	23 (29)	12 (30)	11 (27)
No. of sleepers			
< 4	22 (28)	13 (33)	9 (23)
4–6	35 (44)	20 (50)	15 (38)
≥ 7	23 (29)	7 (18)	16 (40)
Firewood use	35 (44)	11 (28)	24 (60)
Insecticide/coil use	11 (14)	0 (0)	11 (28)
Bed net use	78 (98)	40 (100)	38 (95)
Eaves open	63 (79)	27 (68)	36 (90)
Room ceilings	19 (24)	10 (25)	9 (23)
Bushes/tall grass	47 (59)	32 (80)	15 (38)
Habitat count (± SD)	8.6 ± 3.48	10.35 ± 2.80	6.9 ± 3.23
Temperature, °C (± SD)	24.97 ± 1.58	23.74 ± 0.95	26.19 ± 1.05
Total rainfall, mm (± SD)	87.07 ± 35.91	58.26 ± 1.66	105.87 ± 38.04

*Note*: The table shows the characteristics of the data collection households at the baseline (July 2014, first month of data collection for data included in the study) with respect to demographic features, and environmental variables. Data were collected using paper surveys by trained data collectors

**Fig. 2:**
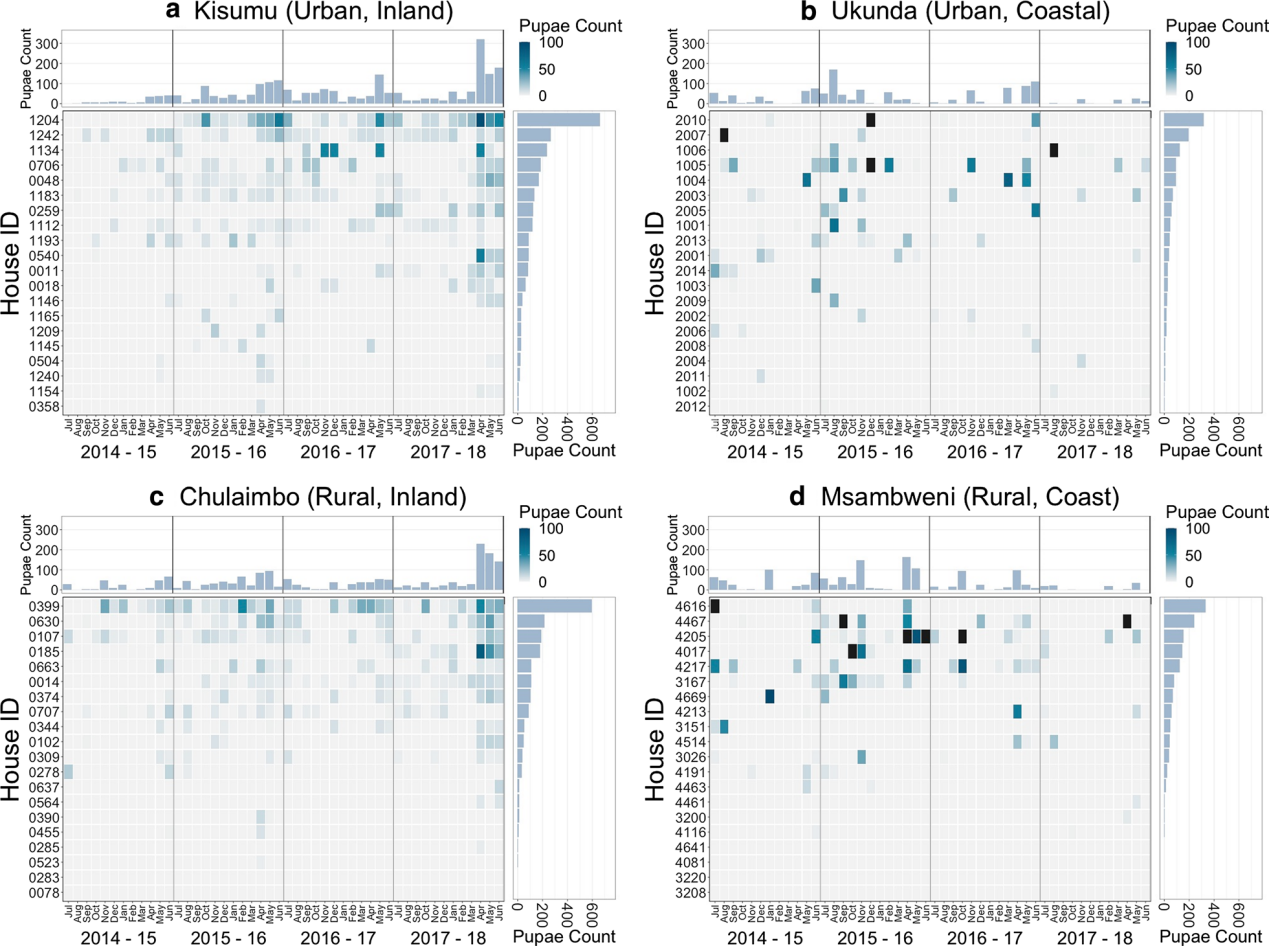
Monthly pupae counts for each household by site. Each study site is represented as a panel: **a** Kisumu, **b** Ukunda, **c** Chulaimbo, **d** Msambweni. For each panel: the main figure (bottom left) shows the number of pupae counted in that month for the given household (darker colors indicate more pupae). The right marginal figure shows the total number of pupae collected from each household in each site over the 4-year data collection period. The top marginal figure shows the total number of pupae collected in each month. Data were collected by trained data collectors who looked around the household and checked breeding containers for any pupae. Pupal persistence within a year (e.g. 2015–2016) was defined as having three or more months in which at least one pupae was found in the household

The total number of *Ae. aegypti* pupae collected across all sites was 9647. Of these 3294 pupae were collected from Msambweni (20 households, rural, coastal site) with a median of 43 (IQR 153) total pupae per household, 2147 pupae were collected from Ukunda (20 households, urban, coastal site) with a median of 54 (IQR 124), 1813 from Chulaimbo (20 households, rural, western site) with a median of 43 (IQR 99), and 2393 from Kisumu (20 households, urban, western site) with a median of 85 (IQR 118) (Fig. 2).

**Fig. 3:**
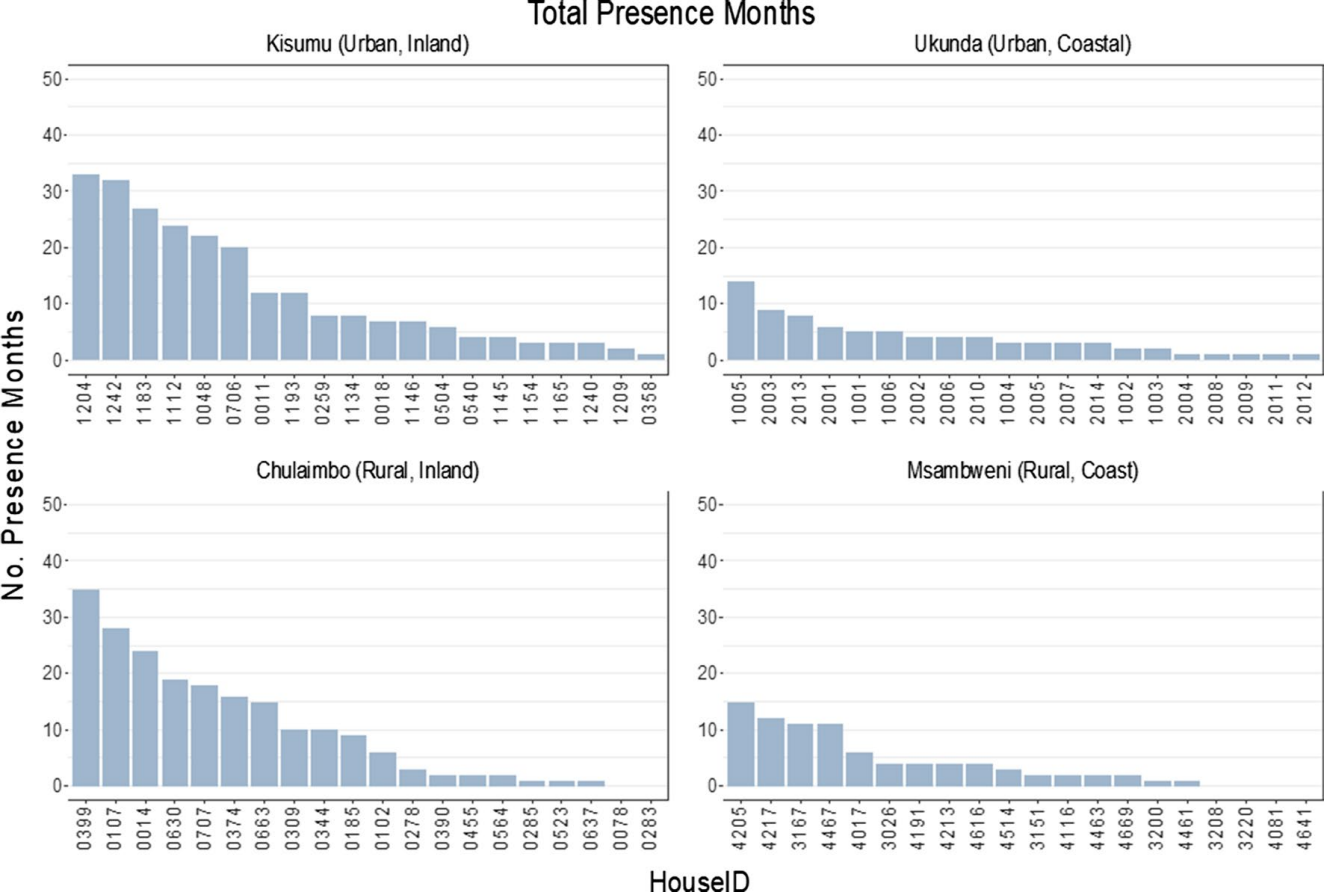
Total number of months of pupal presence over four years by site. The total number of months in which at least one individual pupa was found in a household, in each site is shown. The total number of months present range from 0 to 35

The median number of months in which pupae were observed in households was 4 and ranged from 0 to 35 (Fig. 3). Of the 80 households sampled 6/80 (7.5%) had no pupae observed throughout the entire study period. Within-year pupal persistence was defined as the presence of any pupae within a household for at least 3 months in the year. We observed 85 house-years of pupal persistence from 40 unique households; 16 from Kisumu, 11 from Chulaimbo, 7 from Ukunda and 6 from Msambweni (Fig. 2). Four households in each of Kisumu and Chulaimbo were persistent for all study years. We found no evidence of spatial autocorrelation from the Moran’s I test on total pupae counts or seasonal pupae counts (Additional file 1: Text S4, Table S2, Table S3).

**Fig. 4:**
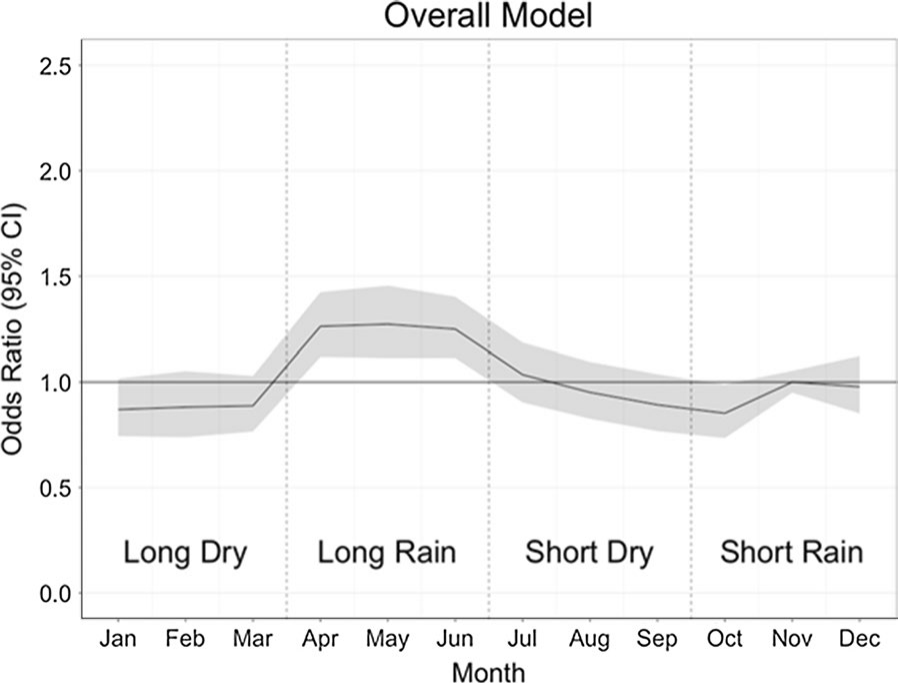
Variation in the risk of increasing pupae abundance by month (non-linear term for month in the abundance models). The four main seasons are shown by dotted lines. The shaded region is the 95% confidence interval

**Fig. 5:**
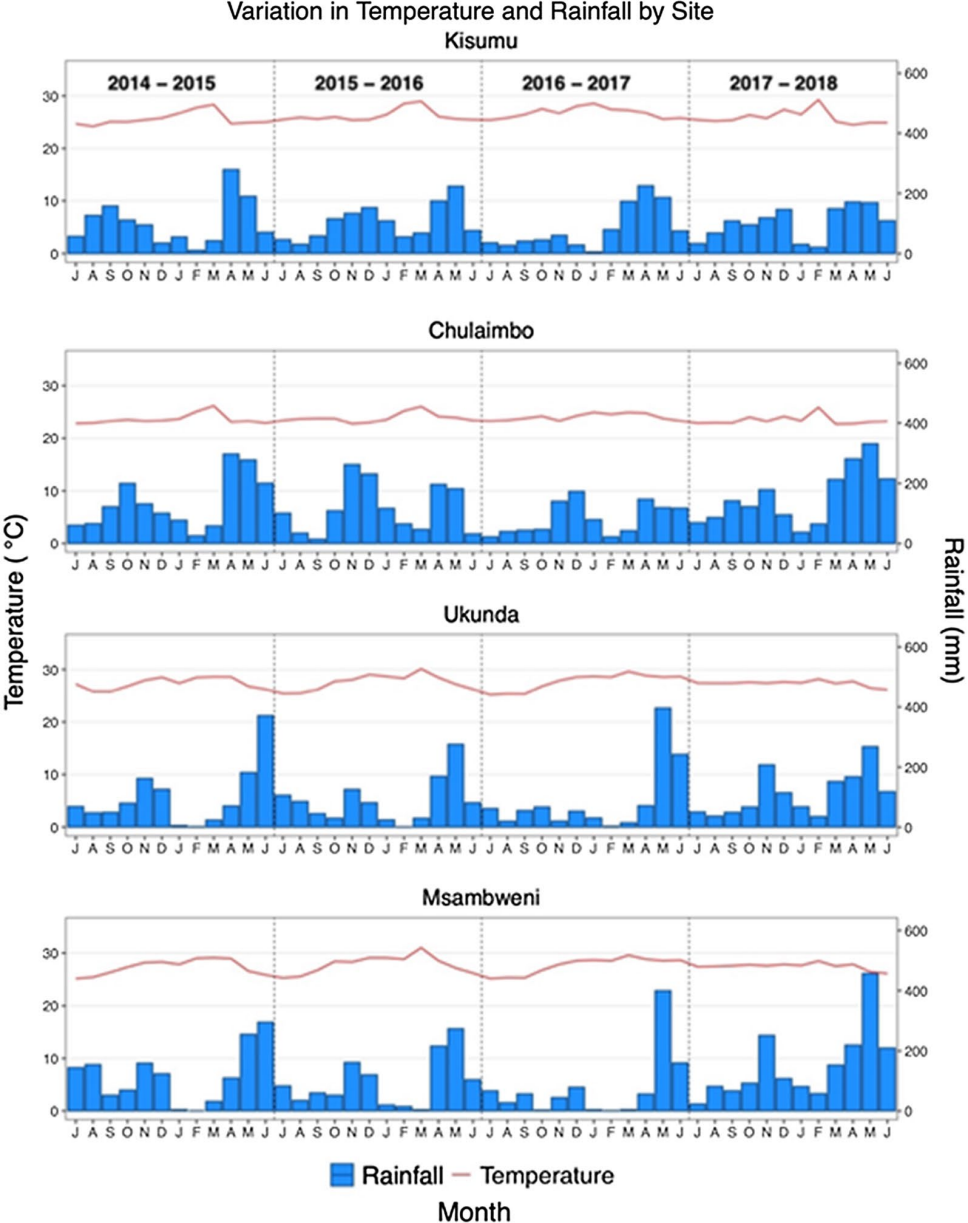
Variation in temperature and rainfall by site for all four years, beginning in July and ending in August of each year. Rainfall and temperature data were collected *via* hobo-loggers at each site. Rainfall data are shown by the blue bars; temperature is shown by the red line

**Fig. 6:**
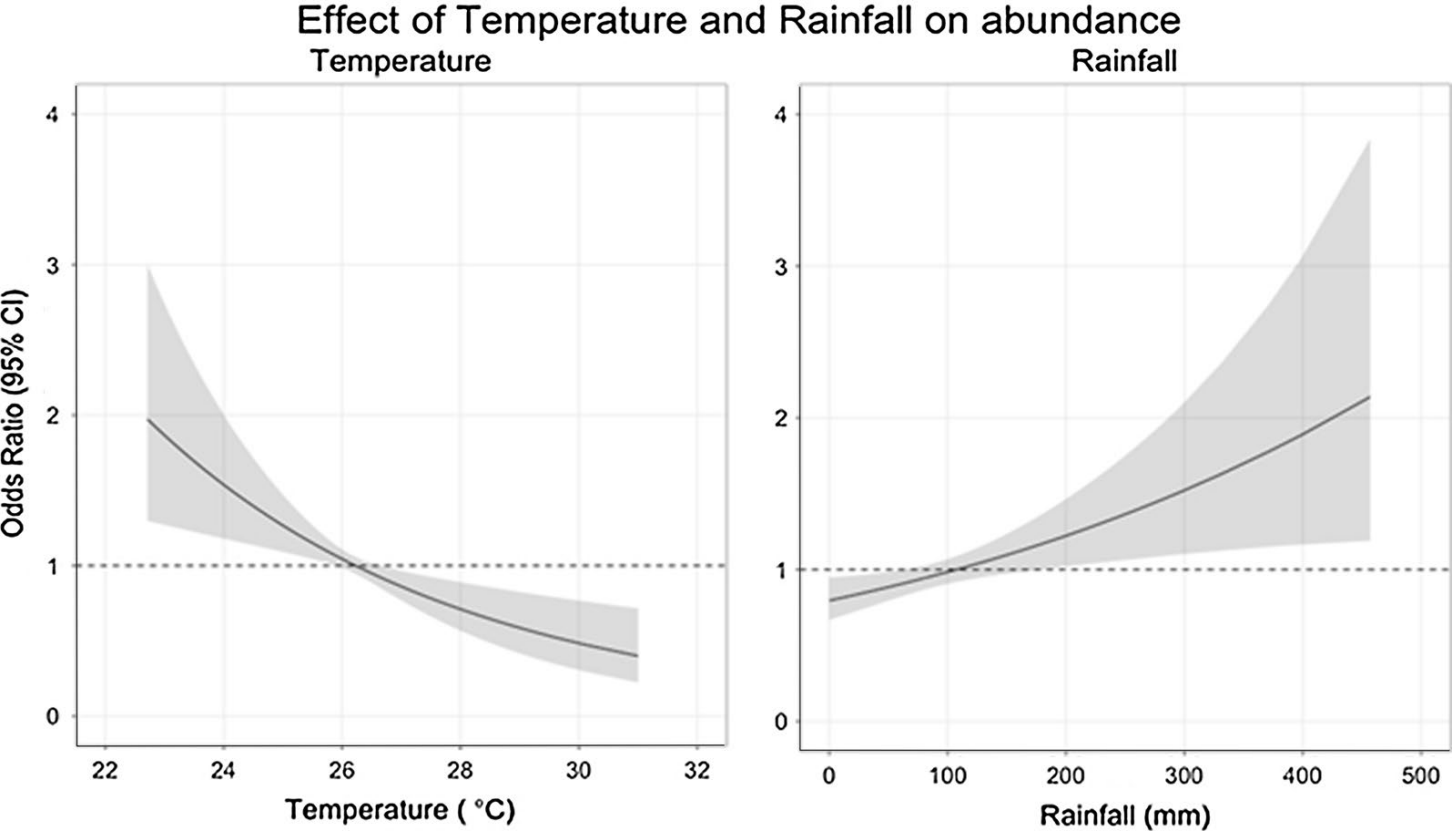
Effect of temperature and rainfall on risk of increasing abundance in the overall pupal abundance model. The shaded region is the 95% confidence interval

**Fig. 7:**
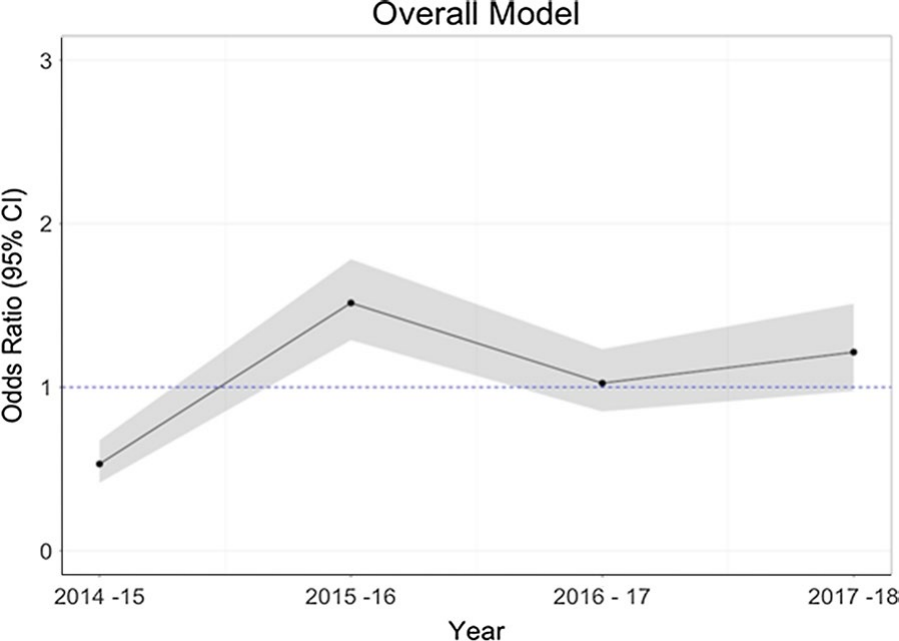
Effect of year of data collection on pupal abundance in the overall model. Year was included as a continuous term in the abundance model (i.e. year 1, 2, 3 and 4). The shaded region is the 95% confidence interval

Tables 3 and 4 show the results of the GAMMs for the risk factors of pupal persistence and abundance. The presence of tall grass or bushes around the houses increases odds of pupal abundance by 60% (OR: 1.60, 95% CI: 1.13–2.28), while presence of eaves (gap between the wall and roof) is associated with a 157% increase in the odds of increasing pupal abundance (OR: 2.57, 95% CI: 1.33–4.95) and increasing habitat counts (breeding containers) are associated with an increase in the odds of pupal abundance (OR: 1.42, 95% CI: 1.21–1.66). Firewood use in a household is associated with a 43% decrease in the odds of pupal abundance (OR: 0.57, 95% CI: 0.37–0.88). The main risk factors for pupal persistence are the presence of bushes/tall grass in the peri-domicile (OR: 4.20, 95% CI: 1.42–12.46) and high habitat counts (OR: 2.17, 95% CI: 1.03–4.58). Figure 4 shows the influence of month on pupal abundance and suggests that risk of pupal abundance due to seasonality is highest during the April-June season (long rainy season) (Additional file 1: Figure S1). Increasing rainfall and decreasing temperature were associated with increasing risk of pupal abundance (Figs. 5, 6). Year of data collection did not have a strong influence on risk of abundance (Fig. 7, Additional file 1: Figure S2). Results for the separate west and coast models are shown in the Additional file 1: Table S4.

**Table 3:** Odds ratios for risk factors for pupal abundance in households in the overall model

House characteristic	Overall model	
	OR	95% CI
Rooms		
< 3	Ref	
3–4	0.94	0.51–1.71
≥ 4	1.01	0.46–2.25
No. of sleepers		
< 4	Ref	
4–6	0.90	0.51–1.60
≥ 7	0.74	0.40–1.36
House wall		
Mud	Ref	
Cement	1.01	0.55–1.85
House roof		
Iron sheet	Ref	
Grass	0.62	0.30–1.25
Tile/asbestos	1.19	0.42–3.38
Room ceilings	1.22	0.61–2.44
Bushes/tall grass	1.60*	1.13–2.28
Firewood use	0.57*	0.37–0.88
Eaves open	2.57**	1.33–4.95
Habitat count	1.42***	1.21–1.66
Insecticide/coil^a^	0.76	0.32–1.77
Location	0.88	0.15–5.11
Urban	1.67	0.31–8.96

*Note*: The table shows the coefficients for the risk factors for pupal abundance in the overall multivariate models

^a^Use of vector control measures. Was a coil or insecticide used in the household

*P < 0.05, **P < 0.01, ***P < 0.001

**Table 4:** Odds ratios for risk factors for pupal persistence in households in the overall model

Characteristic	Overall	
	OR	95% CI
Rooms		
< 3	Ref	–
3–4	0.77	0.21–2.82
≥ 4	1.03	0.17–6.18
No. of sleepers		
< 4	Ref	–
4–6	0.91	0.24–3.51
≥ 7	0.49	0.10–2.46
House wall		
Mud	Ref	–
Cement	1.18	0.27–5.06
House roof		
Iron sheet	Ref	–
Grass	1.81	0.28–11.73
Asbestos/tile	1.28	0.14–11.82
Room ceilings	0.93	0.22–3.86
Bushes/tall grass	4.20*	1.42–12.46
Firewood use	0.57	0.20–1.65
Eaves open	1.66	0.40–6.84
Habitat count	2.17^*^	1.03–4.58
Urban	1.48	0.42–5.22
West	3.44	0.53–22.25

*Note*: The table shows the coefficients for the risk factors for pupal persistence in the overall multivariate models

*P < 0.05, **P < 0.01, ***P < 0.001

## Discussion

*Aedes aegypti* is an anthropophilic, urban mosquito species, and a vector for several arboviruses of public health concern. Understanding the influence of human factors on vector abundance is crucial to planning effective vector control interventions. In this study we use demographic, environmental and entomological data to evaluate the hypothesis that pupal productivity is driven by a small subset of households that exhibit repeated infestation or pupal persistence, and also evaluate the risk factors for pupal persistence and abundance. In this study we report the existence of households that exhibit pupal persistence and identify vegetation in the peri-domicile area and high counts of breeding containers as the main risk factors for pupal persistence.

The use of *Stegomyia* indices and surveillance of immatures, as proxies for adult abundance in *Ae. aegypti* vector ecology is well established [55–57]. While these measures can be useful, they do not account for other epidemiologically important factors and fail to accurately correlate with disease risk [22, 58, 59]. This study establishes the existence of specific household premises that are repeatedly infested with pupae, i.e. persistent households. While pupal persistence or repeated infestation has received little attention in the literature, it may provide a more precise measure of vector abundance. Further, numerous studies have evaluated the influence of environmental and human risk factors on pupal presence and abundance [19, 20, 28]. We found that while persistence and abundance are two different measures, their risk factors are largely similar. Houses that consistently have high pupal counts can be considered key premises and possible super spreader premises and they may play an important role in maintaining vector populations in the study areas. Identifying households with pupal persistence can inform precise and targeted vector control efforts, which would maximize efficiency with limited resources compared to blanket interventions.

Vegetation around houses, high counts of breeding containers and presence of open eaves were significantly associated with risk of pupal abundance. Majority of households (79%) in our study sites had open eaves. House designs with open eaves are preferred as a means to regulate house temperature by promoting air circulation, since the region is characterized by hot and humid conditions particularly at the coast. Moreover, use of wood fuel in some households makes it necessary to have open eaves for adequate ventilation. However, open eaves can be exploited by female mosquitoes to access human hosts and oviposition sites in and around the houses. Since *Ae. aegypti* is a known endophilic and anthropophagic species that can also be found resting around human dwellings [11, 60–62] and remains close to breeding sites [45, 46, 63], houses with open eaves and suitable breeding sites are ideal for their development and survival. Although improvement in house designs to include closed eaves plays a critical role in preventing house entry by malaria vectors [64–67], the role of open eaves on house entry and exit behavior by *Ae. aegypti* which is principally a day-biting mosquito has received little attention. The results of this study suggest that houses with closed eaves may play an important role in regulating *Ae. aegypti* house entry and exit behavior particularly in houses with screened doors and windows that are recognized as main entry points for the culicine mosquitoes [68, 69]. Closed eaves may have an impact on *Ae. aegypti* breeding activity by limiting access to potential breeding sites and human hosts. *Aedes aegypti* obtain blood meals mostly from people inside a given household [70] where they may also find resting places after blood feeding [61]. Gravid mosquitoes exploit indoor and outdoor wet containers for oviposition, hence the need for house entry and exit routes which may include open eaves [68].

The presence of vegetation such as bushes or tall grass in the area around the household is also a strong risk factor for both pupal abundance and persistence. Two of our study sites are located in a rural setting where presence of vegetation in the peri-domicile environment is common due to small scale farming practices that promote vegetation growth in the proximity of houses. This coupled with inadequate environmental hygiene practices in some of the urban households especially those located in the low income-unplanned settlements, contribute to the occurrence of vegetation around households. In this region unplanned urban settlements are characterized by poor hygiene, inadequate water, sewer and waste management systems. Investigations have shown that micro-environmental conditions such as those provided by locations sheltered from sunlight affect the suitability of wet containers as breeding sites for *Ae. aegypti* [28, 71]. Vegetation in proximity to potential breeding containers increases the suitability of breeding containers for infestation by providing shade and decreasing rates of evaporation. Although temperature has been considered as the primary driver of development and survival of mosquito immatures [72], water in containers under direct exposure to sunlight may reach temperatures that are lethal to *Ae aegypti* immatures. Water temperatures above 35 °C have significant impact on larval development [73, 74]. In addition, vegetation contributes organic nutrients for aquatic organisms such as larvae which feed on aquatic microorganisms and provides resting sites for adult mosquitoes. Previous work has found that presence of trees or organic matter results in better survival and faster development of larvae and pupae [21, 74].

Temporal variation in pupal abundance within households across months was consistent with several other studies [20, 28, 29, 75]. Peak pupal abundance was observed in the months of April-June, coinciding with the long rainy season. Climatic variables such as rainfall, humidity and temperature are strongly associated with transmission of arboviruses due to their effect on vector abundance [76–78]. Heavy rainfall appears to favor a rapid increase in the abundance of mosquito vectors and may also extend the transmission period of arboviruses [79, 80]. In addition to providing ideal climatic conditions for vector breeding, heavy rainfall is associated with proliferation of rain fed artificial and natural breeding containers [12, 79]. We also observe a slight increase in the abundance of pupae from 2014–2018 (Fig. 7, Additional file 1: Figure S2). This increase is stronger in the western sites than the coastal sites. This trend suggests that there may be factors increasing the suitability of these sites for pupal productivity over time. Further work is required to identify what factors may be acting and what interventions would work best to stop or reverse the trend.

The presence of large numbers of potential breeding containers is a strong risk factor for pupal abundance and persistence. This relationship is well documented for both *Aedes* and *Anopheles* mosquitoes [38, 81–83]. Potential breeding containers accumulate as a result of human activities within the domestic environment such as water storage and management of solid waste [33]. Due to inadequate water supply systems in most households in our study sites, storage of water in diverse containers is common. This coupled with poor management of solid waste promote the proliferation of potential breeding containers for *Ae. aegypti*. Management of containers has been found to be highly variable in time and space depending on their function, several interventions focus on reducing the number of containers available for breeding. Factors contributing to the infestation of containers with *Aedes* immatures, such as shade and water temperature, have also been examined [28, 74, 84]. Reducing breeding containers remains one of the most important general vector control interventions available.

Firewood use was found to have a protective effect on pupal abundance. Use of wood fuel is common in poor households in rural areas and low income-urban settlements as observed in some households in our study sites. Smoke from domestic fuels may have repellent effect on mosquitoes however, it may not provide effective protection against mosquitoes and has been linked to some health concerns due to indoor air pollution [85].

Patterns of pupal persistence were found to differ across the different sites with the two inland sites having more persistent households than the two coastal sites. The effect of location in our model (inland *vs* coast) was also relatively high, though not statistically significant. This suggests that there may be some important differences between the inland and coastal locations that contribute to pupal persistence that may warrant further investigation. However, inland and coastal sites included in our study generally exhibit different patterns of dengue endemicity with the coastal region being endemic to dengue since 1982 [86]. Moreover, in this study more pupae (56%) were collected from coastal sites than in the western sites (44%), a finding that is consistent with a recent study in the region [12].

Pupal abundance and persistence did not exhibit any spatial correlation. Previous work has shown that pupal productivity in premises has highly focal spatial correlation within approximately 30 m [41]. This corresponds to the known *Ae. aegypti* range of about 50–100 m. The households included in our study were relatively sparsely distributed (more than 100 m apart), and this may be why we observe no spatial effect on total pupae counts or seasonal pupae counts (Additional file 1: Text S4, Tables S3, S4). To identify spatial correlation of productivity and potential productivity hotspots, a more precise study including large numbers of adjacent households (within 30–50 m of one another) would need to be conducted.

### Limitations

Several other important risk factors for pupal productivity have been examined including building materials and density of occupants. While these variables are included in our model, their effects did not reach statistical significance. Due to the documented effects of these variables this may be a result of the low power of our study to detect these effects. In addition, while data collectors attempted to always include the same house in the surveys, certain houses were only available at certain time points. Missing houses were replaced with houses in close proximity (Additional file 1: Text S1). We conducted a sensitivity analysis to evaluate the influence of these household replacements and find that they do not severely alter our main results (Additional file 1: Table S5). The presence of missing data points is also a limitation of our analysis. Missing predictor data were dealt with by simple imputation.

Households included in our study sample were randomly selected from a census enumeration list. While this reduced bias in inclusion of households in our study, the sample may still not be representative. This is particularly true if urban areas have a larger number of households than rural areas. Future work should sample households based on the underlying population distributions. The larger study sites are themselves not representative of rural/urban areas in Kenya but were chosen to include a wide range of environmental and socio-demographic localities (urban/rural, inland/coastal, etc.). This allows us to control for any underlying influence these factors have on abundance or persistence. To specifically examine the influence of these larger scale geographical factors a wider range of areas would need to be sampled.

## Conclusions

Efficient vector management of *Ae. aegypti* is vital to the control and management of arboviruses in endemic areas. In this study we show the existence of pupal persistence in a subset of households in rural and urban Kenya. High counts of potential breeding containers, vegetation in the peri-domicile area and presence of open eaves were strongly associated with increased risk of pupal abundance and persistence, while firewood use was protective against high pupae counts. Our results suggest that targeting households that exhibit pupal persistence in vector control interventions may result in more efficient use of limited resources. Furthermore, vector control interventions should target risk factors for abundance such as vegetation in the peri-domicile area in addition to current interventions involving reducing the numbers of breeding habitats.

## Supplementary information

**Additional file 1: Text S1.** Models and sensitivity analyses: proportional odds pupal abundance models, logistic regression pupal persistence models and sensitivity analyses of household replacements. **Text S2.** Survey data collection forms for immature and adult mosquitoes**. Text S3.** Entomological surveillance protocols: larval and adult mosquito sampling standard operating procedures (SOPs). **Text S4.** Global Moran’s I statistic for evaluating spatial autocorrelation of the total number of pupae observed in the households. **Figure S1.** Effect of season on pupal abundance in the overall model and, in the inland (western) and coastal models. **Figure S2**. The influence of year on pupal abundance in the overall model, and in the inland and coastal models. **Table S1.** Comparison of model fit between alternate models using AIC/BIC/GCV in the pupae abundance model. **Table S2**. Spatial autocorrelation of household pupae count using Moran’s I statistic. **Table S3.** Evaluating within season spatial autocorrelation of pupal abundance in households for the 4 seasons. **Table S4.** Comparison of risk factors for increased pupal abundance in coastal and western household models with complete model**. Table S5.** Sensitivity analyses results: Risk factors for pupal abundance and persistence after excluding household replacements from the overall model.

## Data Availability

Data supporting the conclusions of this article are provided within the article and its additional file. The datasets used and analyzed during the present study are available from the corresponding author on reasonable request.

## Abbreviations

GAMM: Generalized additive mixed model
OR: Odds ratio
CI: Confidence interval

## References

[1] Saifur RG, Dieng H, Hassan AA, Salmah MRC, Satho T, Miake F (2012). Changing domesticity of *Aedes aegypti* in northern peninsular Malaysia: reproductive consequences and potential epidemiological implications. PLoS ONE..

[2] Agha SB, Chepkorir E, Mulwa F, Tigoi C, Arum S, Guarido MM (2017). Vector competence of populations of *Aedes aegypti* from three distinct cities in Kenya for chikungunya virus. PLoS Negl Trop Dis..

[3] Bhatt S, Gething P, Brady O, Messina J, Farlow AW, Moyes CL (2013). The global distribution and burden of dengue. Nature..

[4] Akhwale W. Dengue fever outbreak response. The East Africa Public Health Lab Network Newsletter, Quarterly Bulletin, Kenya chapter. Vol. 7. Nairobi: Department of Disease Prevention and Control, MOH; 2013. p. 1–2.

[5] Ellis E, Neatherlin J, Delorey M, Ochieng M, Mohamed A, Mogeni D (2015). A household serosurvey to estimate the magnitude of a dengue outbreak in Mombasa, Kenya, 2013. PLoS Negl Trop Dis..

[6] Sergon K, Njuguna C, Kalani R, Ofula V, Onyango C, Konongoi L (2008). Seroprevalence of chikungunya virus (CHIKV) infection on Lamu Island, Kenya, October 2004. Am J Trop Med Hyg.

[7] Vu D, Banda T, Teng C, Heimbaugh C, Muchiri E, Mungai P (2017). Dengue and West Nile virus transmission in children and adults in coastal Kenya. Am J Trop Med Hyg..

[8] Chikungunya - Kenya. http://who.int/csr/don/09-august-2016-chikungunya-kenya/en/. Accessed 8 Feb 2019.

[9] Chikungunya - Mombasa Kenya. https://www.who.int/csr/don/27-february-2018-chikungunya-kenya/en/. Accessed 8 Feb 2019.

[10] Lutomiah J, Barrera R, Makio A, Mutisya J, Koka H, Owaka S (2016). Dengue outbreak in Mombasa city, Kenya, 2013–2014: entomologic investigations. PLoS Negl Trop Dis..

[11] Ndenga B, Mutuku F, Ngugi H, Mbakaya J, Aswani P, Musunzaji P (2017). Characteristics of *Aedes aegypti* adult mosquitoes in rural and urban areas of western and coastal Kenya. PLoS ONE..

[12] Ngugi H, Mutuku F, Ndenga B, Musunzaji P, Mbakaya J, Aswani P (2017). Characterization and productivity profiles of *Aedes aegypti* (L.) breeding habitats across rural and urban landscapes in western and coastal Kenya. Parasit Vectors..

[13] Trpis M (1972). Seasonal changes in the larval populations of *Aedes aegypti* in two biotopes in Dar es Salaam, Tanzania. Bull World Health Organ..

[14] McBride CS, Baier F, Omondi AB, Spitzer SA, Lutomiah J, Sang R (2014). Evolution of mosquito preference for humans linked to an odorant receptor. Nature..

[15] Monteiro FJC, Mourão FRP, Ribeiro ESD, Rêgo M, Frances P, Souto RNP (2020). Prevalence of dengue, Zika and chikungunya viruses in *Aede*s (*Stegomyia*) *aegypti* (Diptera: Culicidae) in a medium-sized city, Amazon, Brazil. Rev Inst Med Trop Sao Paulo..

[16] Morales-Pérez A, Nava-Aguilera E, Balanzar-Martínez A, Cortés-Guzmán A, Gasga-Salinas D, Rodríguez-Ramos I (2017). *Aedes aegypti* breeding ecology in Guerrero: cross-sectional study of mosquito breeding sites from the baseline for the Camino Verde trial in Mexico. BMC Publ Health..

[17] Wongkoon S, Jaroensutasinee M, Jaroensutasinee K, Preechaporn W (2007). Development sites of *Aedes aegypti* and *Ae. albopictus* in Nakhon Si Thammarat, Thailand. Dengue Bull..

[18] Jiménez-Alejo A, Morales-Pérez A, Nava-Aguilera E, Flores-Moreno M, Apreza-Aguilar S, Carranza-Alcaraz W (2017). Pupal productivity in rainy and dry seasons: findings from the impact survey of a randomised controlled trial of dengue prevention in Guerrero, Mexico. BMC Publ Health..

[19] Stewart-Ibarra A, Ryan S, Beltra’n E, Mejı’a R, Silva M, Munoz A (2013). Dengue vector dynamics (*Aedes aegypti*) influenced by climate and social factors in Ecuador: implications for targeted control. PLoS ONE..

[20] Hiscox A, Kaye A, Vongphayloth K, Banks I, Piffer M, Khammanithong P (2013). Risk factors for the presence of *Aedes aegypti* and *Aedes albopictus* in domestic water-holding containers in areas impacted by the Nam Theun 2 Hydroelectric project. Laos. Am J Trop Med Hyg..

[21] Barrera R, Amador M, Clark GG (2006). Ecological factors influencing *Aedes aegypti* (Diptera: Culicidae) productivity in artificial containers in Salinas, Puerto Rico. J Med Entomol..

[22] Focks D, Alexander N. Multicountry study of *Aedes aegypti* pupal productivity survey methodology: findings and recommendations. In: World Health Organization and Special Programme for Research and Training in Tropical Diseases. Geneva, Switzerland: World Health Organization; 2006.

[23] Focks D, Brenner R, Hayes J, Daniels E (2000). Transmission thresholds for dengue in terms of *Aedes aegypti* pupae per person with discussion of their utility insource reduction efforts. Am J Trop Med Hyg..

[24] Wijayanti S, Sunaryo S, Suprihatin S, McFarlane M, Rainey S, Dietrich I (2016). Dengue in Java, Indonesia: relevance of mosquito indices as risk predictors. PLoS Negl Trop Dis..

[25] WHO (2009). Dengue guidelines for diagnosis, treatment, prevention and control.

[26] Sabchareon A, Wallace D, Sirivichayakul C, Limkittikul K, Chanthavanich P, Suvannadabba S (2012). Protective efficacy of the recombinant, live-attenuated, CYD tetravalent dengue vaccine in Thai schoolchildren: a randomised, controlled phase 2b trial. Lancet..

[27] Halstead S (2012). Dengue vaccine development: a 75% solution?. Lancet..

[28] Islam S, Haque C, Hossain E, Rochon K (2019). Role of container type, behavioural, and ecological factors in *Aedes* pupal production in Dhaka, Bangladesh: an application of zero-inflated negative binomial model. Acta Trop..

[29] Paul K, Dhar-Chowdhury P, Haque C, Al-Amin H, Goswami D, Kafi M (2018). Risk factors for the presence of dengue vector mosquitoes, and determinants of their prevalence and larval site selection in Dhaka, Bangladesh. PLoS ONE..

[30] Dhar-Chowdhury P, Emdad Haque C, Lindsay R, Hossain S (2016). Socioeconomic and ecological factors influencing *Aedes aegypti* prevalence, abundance, and distribution in Dhaka, Bangladesh. Am J Trop Med Hyg..

[31] Dom NC, Ahmad AH, Ishak AR, Ismail R (2013). Assessing the risk of dengue fever based on the epidemiological, environmental and entomological variables. Procedia Soc Behav Sci..

[32] Garcia-Sánchez D, Pinilla G, Quintero J (2017). Ecological characterization of *Aedes aegypti* larval habitats (Diptera: Culicidae) in artificial water containers in Girardot. Colombia. J Vector Ecol..

[33] Barrera R, Amador M, MacKay AJ (2011). Population dynamics of *Aedes aegypti* and dengue as influenced by weather and human behavior in San Juan. Puerto Rico. PLoS Negl Trop Dis..

[34] Padmanabha H, Soto E, Mosquera M, Lord C, Lounibos L (2010). Ecological links between water storage behaviors and *Aedes aegypti* production: implications for dengue vector control in variable climates. EcoHealth..

[35] Quintero J, Carrasquilla G, Suárez S, González C, Olano V (2009). An ecosystemic approach to evaluating ecological, socioeconomic and group dynamics affecting the prevalence of *Aedes aegypti* in two Colombian towns. Cad Saúde Pública..

[36] Hayden M, Uejio C, Rosales C, Walker K, Ramberg F (2010). Microclimate and human factors in the divergent ecology of *Aedes aegypti* along the Arizona, US/Sonora, MX Border. EcoHealth..

[37] Tun-Lin W, Kay B, Barnes A (1995). The premise condition index: a tool for streamlining surveys of *Aedes aegypti*. Am J Trop Med Hyg..

[38] Garelli F, Espinosa M, Gurtler R (2013). Spatial analysis of *Aedes aegypti* immatures in northern Argentina: clusters and temporal instability. Acta Trop..

[39] Koenraadt C, Aldstadt J, Kijchalao U, Sithiprasasna R, Getis A, Johns J (2008). Spatial and temporal patterns in pupal and adult production of the dengue vector *Aedes aegypti* in Kamphaeng Phet, Thailand. Am J Trop Med Hyg..

[40] Schafrick N, Milbrath M, Berrocal V, Wilson M, Eisenberg J (2013). Spatial clustering of *Aedes aegypti* related to breeding container characteristics in coastal Ecuador: implications for dengue control. Am J Trop Med Hyg..

[41] LaCon G, Morrison A, Astete H, Stoddard S, Paz-Soldan V, Elder J (2014). Shifting patterns of *Aedes aegypti* fine scale spatial clustering in Iquitos, Peru. PLoS Negl Trop Dis..

[42] Waller L, Gotway C (2004). Applied spatial statistics for public health data.

[43] Shah MM, Krystosik AR, Ndenga BA, Mutuku FM, Caldwell JM, Otuka V (2019). Malaria smear positivity among Kenyan children peaks at intermediate temperatures as predicted by ecological models. Parasit Vectors..

[44] Getis A, Morrison AC, Gray K, Scott TW (2003). Characteristics of the spatial pattern of the dengue vector, *Aedes aegypti*, in Iquitos, Peru. Am J Trop Med Hyg..

[45] Harrington L, Scott T, Lerdthusnee K, Coleman R, Costero A, Clark G (2005). Dispersal of the dengue vector *Aedes aegypti* within and between rural communities. Am J Trop Med Hyg..

[46] Trpis M, Hausermann W (1986). Dispersal and other population parameters of *Aedes aegypti* in African village and their possible significance in epidemiology of vector-borne diseases. Am J Trop Med Hyg..

[47] Wood S (2017). Generalized additive models an introduction with R.

[48] Guisan A, Harrell F (2000). Ordinal response regression models in ecology. J Veg Sci..

[49] Harrell F (2001). Regression modeling strategies.

[50] Brezger A, Kneib T, Lang S (2005). BayesX: analyzing Bayesian structural additive regression models. J Stat Softw..

[51] Kneib T, Fahrmeir L, Richard E, Chandler E, Scott M (2011). A space-time study on forest health. Statistical methods for trend detection and analysis in the environmental science.

[52] Umlauf N, Adler D, Kneib T, Lang S, Zeileis A (2015). Structured additive regression models: an R Interface to BayesX. J Stat Softw..

[53] R Core Development Team. R: A language and environment for statistical computing. 3.5.1 edition. Vienna, Austria: R Foundation for Statistical Computing; 2018.

[54] Bivand RS, Wong DWS (2018). Comparing implementations of global and local indicators of spatial association. TEST..

[55] Chadee DD, Huntley S, Focks DA, Chen AA (2009). *Aedes aegypti* in Jamaica, West Indies: container productivity profiles to inform control strategies. Trop Med Int Health..

[56] Getachew F, Moges T, Ebba A, Jemberie K, Yitayih W, Demekech D (2018). Distribution and larval breeding habitats of *Aedes* mosquito species in residential areas of northwest Ethiopia. Epidemiol Health..

[57] Troyo A, Calderón-Arguedas O, Fuller DO, Solano ME, Avendaño A, Arheart KL (2008). Seasonal profiles of *Aedes aegypti* (Diptera: Culicidae) larval habitats in an urban area of Costa Rica with a history of mosquito control. J Vector Ecol..

[58] Chadee D (2004). Key premises, a guide to *Aedes aegypti* (Diptera: Culicidae) surveillance and control. Bull Entomol Res.

[59] Focks D, Chadee D (1997). Pupal survey: an epidemiologically significant surveillance method for *Aedes aegypti*: an example using data from Trinidad. Am J Trop Med Hyg..

[60] Scott TW, Amerasinghe E, Morrison A, Lorenz L, Clark GG, Strickman D (2000). Longitudinal studies of *Aedes aegypti* (Diptera: Culicidae) in Thailand and Puerto Rico: blood feeding frequency. J Med Entomol.

[61] Chadee DD (2013). Resting behaviour of *Aedes aegypti* in Trinidad: with evidence for the re-introduction of indoor residual spraying (IRS) for dengue control. Parasit Vectors..

[62] Perich M, Davila G, Turner A, Garcia A, Nelson M (2000). Behavior of resting *Aedes aegypti* (Diptera: Culicidae) and its relation to ultra-low volume adulticide efficacy in Panama City, Panama. J Med Entomol..

[63] Bergero P, Ruggerio C, Lombardo R, Schweigmann N, Solari H (2013). Dispersal of *Aedes aegypti:* field study in temperate areas using a novel method. J Vector Borne Dis..

[64] Ogoma S, Lweitoijera D, Ngonyani H, Furer B, Russell T, Mukabana W (2010). Screening mosquito house entry points as a potential method for integrated control of endophagic filariasis, arbovirus and malaria vectors. PLoS ONE..

[65] Mburu M, Juurlink M, Spitzen J, Moraga P, Hiscox A, Mzilahowa T (2018). Impact of partially and fully closed eaves on house entry rates by mosquitoes. Parasit Vectors..

[66] Menger S, Omusula P, Wouters K, Oketch C, Carreira A, Durka M (2016). Eave screening and push-pull tactics to reduce house entry by vectors of malaria. Am J Trop Med Hyg..

[67] Jatta E, Jawara M, Bradley J, Jeffries D, Kandeh B, Knudsen J (2018). How house design affects malaria mosquito density, temperature, and relative humidity: an experimental study in rural Gambia. Lancet Planet Health..

[68] Njie M, Dilger E, Lindsay S, Matthew J (2009). Importance of eaves to house entry by anopheline, but not culicine, mosquitoes. J Med Entomol..

[69] Che-Mendoza A, Medina-Barreiro A, Koyoc-Cardeña E, Uc-Puc V, Contreras-Perera Y, Herrera-Bojorquez J (2018). House screening with insecticide-treated netting provides sustained reductions in domestic populations of *Aedes aegypti* in Merida, Mexico. PLoS Negl Trop Dis..

[70] De Benedictis J, Chow-Shaffer E, Costero A, Clark GG, Edman JD, Scott TW (2003). Identification of the people from whom engorged *Aedes aegypti* took blood meals in Florida, Puerto Rico, using polymerase chain reaction-based DNA profiling. Am J Trop Med Hyg..

[71] Vezzani D, Schweigmann N (2002). Suitability of containers from different sources as breeding sites of *Aedes aegypti* (L.) in a cemetery of Buenos Aires City, Argentina. Mem Inst Oswaldo Cruz..

[72] Couret J, Dotson E, Benedict M (2014). Temperature, larval diet, and density effects on development rate and survival of *Aedes aegypti* (Diptera: Culicidae). PLoS ONE..

[73] Farjana T, Tuno N, Higa Y (2012). Effects of temperature and diet on development and interspecies competition in *Aedes aegypti* and *Aedes albopictus*. Med Vet Entomol..

[74] Tun-Lin W, Burkot TR, Kay BH (2000). Effects of temperature and larval diet on development rates and survival of the dengue vector *Aedes aegypti* in north Queensland, Australia. Med Vet Entomol..

[75] Midega JT, Nzovu J, Kahindi S, Sang RC, Mbogo C (2006). Application of the pupal/demographic-survey methodology to identify the key container habitats of *Aedes aegypti* (L.) in Malindi district, Kenya. Ann Trop Med Parasitol..

[76] Goulda E, Higgs S (2009). Impact of climate change and other factors on emerging arbovirus diseases. Trans R Soc Trop Med Hyg..

[77] Dhimal M, Gautam I, Joshi H, O’Hara R, Ahrens B, Kuch U (2015). Risk factors for the presence of chikungunya and dengue vectors (*Aedes aegypti* and *Aedes albopictus*), their altitudinal distribution and climatic determinants of their abundance in Central Nepal. PLoS Negl Trop Dis..

[78] Barrera R, Amador M, MacKay A (2011). Population dynamics of *Aedes aegypti* and dengue as influenced by weather and human behavior in San Juan, Puerto Rico. PLoS Negl Trop Dis..

[79] Agha SB, Tchouassi DP, Bastos ADS, Sang R (2017). Dengue and yellow fever virus vectors: seasonal abundance, diversity and resting preferences in three Kenyan cities. Parasit Vectors..

[80] Roiz D, Boussès P, Simard F, Paupy C, Fontenille D (2015). Autochthonous chikungunya transmission and extreme climate events in southern France. PLoS Negl Trop Dis..

[81] Li Y, Kamara F, Zhou G, Puthiyakunnon S, Li C, Yan G (2014). Urbanization increases *Aedes albopictus* larval habitats and accelerates mosquito development and survivorship. PLoS Negl Trop Dis..

[82] Nguyen LAP, Clements ACA, Jeffery JAL, Yen NT, Nam VS, Vaughan G (2011). Abundance and prevalence of *Aedes aegypti* immatures and relationships with household water storage in rural areas in southern Viet Nam. Int Health..

[83] Zahouli JBZ, Koudou BG, Müller P, Malone D, Tano Y, Utzinger J (2017). Urbanization is a main driver for the larval ecology of *Aedes* mosquitoes in arbovirus-endemic settings in south-eastern Côte d’Ivoire. PLoS Negl Trop Dis..

[84] Overgaard HJ, Olano VA, Jaramillo JF, Matiz MI, Sarmiento D, Stenström TA (2017). A cross-sectional survey of *Aedes aegypti* immature abundance in urban and rural household containers in central Colombia. Parasit Vectors..

[85] Biran A, Smith L, Lines J, Ensink J, Cameron M (2007). Smoke and malaria: are interventions to reduce exposure to indoor air pollution likely to increase exposure to mosquitoes?. Trans R Soc Trop Med Hyg..

[86] Johnson BK, Ocheng D, Gichogo A, Okiro M, Libondo D, Kinyanjui P (1982). Epidemic dengue fever caused by dengue type 2 virus in Kenya: preliminary results of human virological and serological studies. East Afr Med J..

