# Supplementary material for: Risk factors for Aedes aegypti household pupal persistence in longitudinal entomological household surveys in urban and rural Kenya
Source: Parasit Vectors. 2020 Oct 1;13:499. doi: 10.1186/s13071-020-04378-7 (PMC7528257; doi:10.1186/s13071-020-04378-7)
Supplement: Supplementary file 1 — Additional file 1: Text S1. Models and sensitivity analyses: proportional odds pupal abundance models, logistic regression pupal persistence models and sensitivity analyses of household replacements. Text S2. Survey data collection forms for immature and adult mosquitoes. Text S3. Entomological surveillance protocols: larval and adult mosquito sampling standard operating procedures (SOPs). Text S4. Global Moran’s I statistic for evaluating spatial autocorrelation of the total number of pupae observed in the households. Figure S1. Effect of season on pupal abundance in the overall model and, in the inland (western) and coastal models. Figure S2. The influence of year on pupal abundance in the overall model, and in the inland and coastal models. Table S1. Comparison of model fit between alternate models using AIC/BIC/GCV in the pupae abundance model. Table S2. Spatial autocorrelation of household pupae count using Moran’s I statistic. Table S3. Evaluating within season spatial autocorrelation of pupal abundance in households for the 4 seasons. Table S4. Comparison of risk factors for increased pupal abundance in coastal and western household models with complete model. Table S5. Sensitivity analyses results: Risk factors for pupal abundance and persistence after excluding household replacements from the overall model. [file 13071_2020_4378_MOESM1_ESM.doc]

**Additional file 1**

**Text S1: Models and Sensitivity Analyses.**

*Proportional Odds Pupal Abundance Models*

We modeled the risk factors for pupal abundance in households using a spatially explicit, longitudinal generalized additive model, with a proportional odds model framework [1-3]. The outcome of interest is pupal abundance category, defined as zero (no pupae observed), low (0 – 15 pupae observed), intermediate (15 – 30 pupae observed) or high (> 30 pupae observed). Given a set of *N* households, each sampled at *T* timepoints, the observed outcome for a household at a given timepoint is *y_it_,* where *i = 1, . . ., N* (N = 80)*, t = 1, . . ., T* (T = 48 months)*,* and takes 1 of *J* values (J = zero, low, intermediate or high). The probability of being in a given abundance category *j* or higher at each time point is modeled using a proportional odds model as follows;


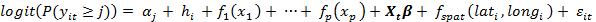


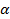
 represents the intercept corresponding to each of the *J* outcomes and *h* represents the household intercept (i.e. random effect for households). The terms
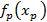
 represent one of *P* non-linear effects, which are modeled using smoothing splines in additive models [3]. The design matrix ***X*** and vector
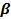
represent the linear terms of interest and their corresponding coefficients. The models include a spatial term
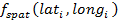
, to account for any spatial correlation [4]. We used geo-splines and the recorded longitude and latitude values of each household to account for spatial correlation. The
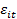
 term represents the independent and identically distributed errors. Household characteristics (use of firewood, presence of eaves, number of occupants, etc.) were modeled as linear terms, while seasonal and temporal effects (month, year, temperature, rainfall) were included in the model as non-linear terms to account for seasonality and any shifts in abundance patterns by year. Models were fit using Restricted Maximum Likelihood estimation in BayesX via its R language interface R2BayesX [4, 5].

For the abundance models, we ran an ‘overall’ model that included data from both the coastal and the inland sites and included a fixed effect to account for Location (Coast vs. Inland). We ran additional abundance models using data from the inland, and the coastal sites separately to evaluate any differences between the two locations. Results are shown in the supplementary materials (Table S1). In addition to the primary model that included month as the seasonal term, we ran models that included temperature and rainfall as seasonal terms. The results of these alternative model structures are reported in the supplementary materials (Table S2). We used residual analyses to evaluate model assumptions (e.g. proportional odds) and identify any outliers. Model choice was based on model fit statistics [1, 2].

*Logistic Regression Pupal Persistence Models*

The risk factors for pupal persistence in households were modeled using a spatially explicit, longitudinal generalized additive model with a binomial outcome. Pupal persistence within a household was defined as presence of pupae within that household three months or more within a year. 80 Households were followed for 48 months (four years) and this resulted in 320 house-years of data. The pupal persistence logistic regression is structured as follows:


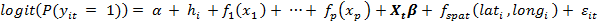


The outcome
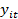
 represents pupal persistence in a household *i, i = 1 . . ., N*, at timepoint *t, t = 1, . . ., 4* (4 years ). The model included a non-linear term for year, as well as a spatial term to account for spatial correlation. The spatial effect was modeled using geo-splines. The
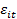
 term represents the independent and identically distributed errors. We built a single model for persistence that included both inland and coastal households due to small sample sizes. We accounted for the Location (coast vs inland) using a fixed effect term and household using a random effect term in the model. Seasonality was not accounted for in the persistence model because persistence is defined by year as opposed to month.

*Sensitivity analyses of household replacements*

Households included in the study at the beginning of the data collection period were sampled randomly from a 2014 census enumeration list. In the subsequent months of data collection, during a routine data collection period, if an originally sampled household was unavailable for data collection an additional house was sampled in its place as a substitute household. In some instances, a household was unavailable for sampling at multiple time points. If this occurred, data collectors made an effort to resample the same substitute household, but this was not always possible. All original households and household replacements were recorded and stored. In the main analysis, in instances were household replacements were conducted, data from the original and substituted houses was merged and the result treated as a single household.

In order to evaluate the influence of household replacements on our results we repeated the regression analyses with only households that had complete data for the entire data collection period and excluded household replacements. Most household replacements occurred in the coastal sites. We re-ran both the persistence and abundance models excluding the replacement households. The results are shown in supplementary Tables S5. Though the household replacement rate is high in Msambweni, Table S5 suggests that the replacements did not have a significant effect on our final results.

**Text S2: Survey data collection forms.**

**i) Aedes immatures data sheets; larvae and pupa.**

**Larval Mosquito Sampling Data Sheet**

**Date: Day____ Month____ Year________ Time …………… Team Leader ……………….**

**Site #……__ House ID ……………………………………**

**INDOOR**

| **Room** | **Container type** | **Container**  **ID** | **Container size** | **species** | **Early**  **instars** | **Late instars** | **pupae** | **Species (after pupal eclosion)** | **Female** | **male** |
| --- | --- | --- | --- | --- | --- | --- | --- | --- | --- | --- |
|  |  |  |  |  |  |  |  |  |  |  |
|  |  |  |  |  |  |  |  |  |  |  |
|  |  |  |  |  |  |  |  |  |  |  |
|  |  |  |  |  |  |  |  |  |  |  |
|  |  |  |  |  |  |  |  |  |  |  |
|  |  |  |  |  |  |  |  |  |  |  |
|  |  |  |  |  |  |  |  |  |  |  |
|  |  |  |  |  |  |  |  |  |  |  |
|  |  |  |  |  |  |  |  |  |  |  |
|  |  |  |  |  |  |  |  |  |  |  |
|  |  |  |  |  |  |  |  |  |  |  |
|  |  |  |  |  |  |  |  |  |  |  |
|  |  |  |  |  |  |  |  |  |  |  |
|  |  |  |  |  |  |  |  |  |  |  |

**OUTDOOR**

| **place** | **Container type** | **Container**  **ID** | **Container size** | **species** | **Early**  **instars** | **Late instars** | **pupae** | **Species (after pupal eclosion)** | **Female** | **male** |
| --- | --- | --- | --- | --- | --- | --- | --- | --- | --- | --- |
|  |  |  |  |  |  |  |  |  |  |  |
|  |  |  |  |  |  |  |  |  |  |  |
|  |  |  |  |  |  |  |  |  |  |  |
|  |  |  |  |  |  |  |  |  |  |  |
|  |  |  |  |  |  |  |  |  |  |  |
|  |  |  |  |  |  |  |  |  |  |  |
|  |  |  |  |  |  |  |  |  |  |  |
|  |  |  |  |  |  |  |  |  |  |  |
|  |  |  |  |  |  |  |  |  |  |  |
|  |  |  |  |  |  |  |  |  |  |  |
|  |  |  |  |  |  |  |  |  |  |  |
|  |  |  |  |  |  |  |  |  |  |  |
|  |  |  |  |  |  |  |  |  |  |  |
|  |  |  |  |  |  |  |  |  |  |  |
|  |  |  |  |  |  |  |  |  |  |  |
|  |  |  |  |  |  |  |  |  |  |  |
|  |  |  |  |  |  |  |  |  |  |  |
|  |  |  |  |  |  |  |  |  |  |  |

Key; 1. Jerry can, 2. Small plastic food container/tin, 3. Bucket, 4. tire, 5.drum, 6.water tanks, 7.domestic animal drinking container, 8.flower pot, 9.vase, 10.cistern, 11.coconut, 12.bottle, 13.sufuria, 14.basin,15 pot, 16, other

Size/capacity; 1.small: <2 liters, 2. Medium: 2 to 7 liters, 3. large: >7 liters

Place: 1. Front yard (FY), 2.bushes around house (BH), 3.dump site (DS) 4.backyard (BY), 5.garden (G)

Room: Bedroom (BD), Sitting room (SR), Corridor (C), Kitchen (K), Bathroom (BR), Toilet (T), Store (S)

**ii) Adult Mosquito Sampling Data Sheet**

**Date: Day____ Month____ Year________ Time …………… Team Leader ……………….**

**Site………………... Site #……__ House ID ……………………………………..**

**INDOORS**

**House wall (Mud=1/Cement=2)..__ House roof (Iron sheets=1/Grass=2)..__**

**Number of rooms ……__ Firewood use in the house Yes=1/No=0)..__**

**Number of sleepers …____ Insecticide sprayed (Yes=1/No=0)..__ Mosquito coil burnt (Yes=1/No=0)..__ Bed net present (Yes=1/No=0)..__ Eaves open (Yes=1/No=0)..__ Rooms with ceilings (Yes=1/No=0)..__**

| **Species** | ***Aedes aegypti*** | ***An. gambiae*** | ***An. funestus*** | ***Culex*** |  |
| --- | --- | --- | --- | --- | --- |
| **Male** |  |  |  |  |  |
| **Un-fed** |  |  |  |  |  |
| **Blood-fed** |  |  |  |  |  |
| **Half-gravid** |  |  |  |  |  |
| **Gravid** |  |  |  |  |  |

**OUTDOORS**

**Bushes around the house (Yes=1/No=0)..__ Tall grass around the house (Yes=1/No=0)..__**

| **Species** | ***Aedes aegypti*** | ***An. gambiae*** | ***An. funestus*** | ***Culex*** |  |
| --- | --- | --- | --- | --- | --- |
| **Male** |  |  |  |  |  |
| **Un-fed** |  |  |  |  |  |
| **Blood-fed** |  |  |  |  |  |
| **Half-gravid** |  |  |  |  |  |
| **Gravid** |  |  |  |  |  |

**Remarks: _______________________________________________________________**

**Text S3: Entomological Surveillance protocols**

1. **Larval sampling SOP**

**1. PURPOSE/APPLICABILITY**

**1.1 Purpose:** To provide guidelines for the procedures to be followed when sampling *Aedes* mosquito larvae and pupae.

**1.2 Applicability:** Entomology and all larval survey field staff.

**2. SUMMARY**

This SOP describes the sampling of immature stages of container breeding mosquitoes of the genus *Aedes.* The survey involves identifying all natural and artificial wet containers in and around houses and examining each of them for larvae and pupae. Larval surveys are traditional methods used for monitoring mosquito populations. They can also be used to monitor the presence, distribution, and density and to determine the efficacy of treatment procedures.

**3. ABBREVIATIONS AND TERMS**

3.1 SOP Standard Operating Procedure.

3.2 Q/A Quality Assurance

**4. RESPOSIBLE PERSONEL**

4.1 Field staff. Ensure adherence to this SOP

4.2 Field supervisor. Ensures that all the field staff adhere to the SOP

**5. EQUIPMNT/MATERIALS**

5.1 Pipettes

5.2 Ladles and plastic larvae rearing trays

5.3 Vials/specimen bottles

5.4 Field data entry forms

5.5 Masking tape

5.6 Pupae emergence paper cups

5.7 Marker pen

5.8 Torches/flashlights

5.9 Cool box

**6. PROCEDURE**

6.1 Field team will carry out larval survey from 0700 to 0100 hours.

6.2 Verbal consent to inspect a house* is sought from each house hold head. When consent is given the field team inspects all the natural and artificial containers in and around the house, including habitats such as tree holes and leaf axils that might harbor *Ae. aegypti* and other mosquitoes to determine whether the containers are wet or dry and whether they contain larvae and/or pupae. Containers located in dark or shaded areas will be inspected using flashlights.

6.3 When the field team is denied access into a household, they move and seek consent in the immediate neighboring household.

6.4 All pupae in wet containers are counted, and together with a sample of larvae are collected using ladles and pipettes, placed in vials, labeled and recorded on standard forms.

6.5 Samples are placed in a cool box and taken to DVBNTD laboratory, where they are counted and identified. Pupae are held in paper cups and allowed to emerge and the adults are identified using appropriate taxonomic keys.

REFERENCES

1. Lenhart, A. E., Castillot, C. E., Oviedo, M. and Villegas. E. (2006).Use of the pupal/demographic-survey technique to identify the epidemiologically important types of containers producing Aedes aegypti (L.) in a dengue-endemic area of Venezuela. *Ann. of Trop. Med. & Para.* 100. Suppl. No. 1, 53-59 (2006).

2. Focks, D.A. and Chadee, D.D. (1997). Pupal survey: An epidemiologically significant surveillance method for *Aedes aegypti*: an example using data from Trinidad.

*Am. J. Trop. Med. Hyg*. 56:159-167.

3. Brown AWA, (1974). Worldwide surveillance of *Aedes aegypti*.*Proc Ann Conf Calif Mosq Control Assoc 42: 20-25*

4. Bang,Y.H, Brown, D.N, Onwubiko AO. (1981). Prevalence of potential yellow fever vectors indomestic water containers in south-east Nigeria. *Bull WHO* 59:107–114

1. **Adult mosquito sampling SOP**

**1. PURPOSE/APPLICABILITY**

**1.1 Purpose:** To provide guidelines for the procedures to be followed in collecting adult live of *Aedes* mosquitoes using battery- operated aspirator (Prokopak)

**1.2 Applicability**: Entomology and mosquito aspiration all field staff.

**2. SUMMARY**

This SOP describes the collection of mosquitoes using the batter-operated aspirator (Prokopack).Method involves mosquito aspirator using the Prokopack collecting mosquitoes resting indoors; on walls and under the roof. The Prokopack is aspirator is a new mosquito sampling tool whose functional design is similar to the CDC backpack aspirator. However, it is much smaller and easier to use, particularly in hard –to- reach areas has been found to be more effective in various scenarios.

**3. ABBREVIATIONS AND TERMS**

3.1 SOP Standard Operating Procedure.

3.2 Q/A Quality Assurance

3.3 DVBND Division of Vector Born and Neglected tropical Diseases

**4. RESPOSIBLE PERSONEL**

4.1 Field staff. Ensure adherence to this SOP

4.2 Field supervisor. Ensures that all the field staff adhere to the SOP

**5. EQUIPMNT/MATERIALS**

**5.1** Prokopack aspirator 5.8 Bottom-meshed tins

5.2 battteries 5.9 Stop watch

5.3 Masking tape/labels

5.4 Marker pen

5.5 Field Data forms

5.6 Bag

5.7 Handle stick

**6. PROCEDURE**

6.1 Houses to be used for mosquito collection are identified and verbal consent sought from the household head prior to exercise.

6.1 A team of two collectors are to collect mosquitoes from 0700 – 1200 hours in selected houses.

6.2 Collectors arrive at the site of collection 30 minutes before starting time set for the collection.

6.3 The collectors assemble the aspirator. That is, connect the Prokopack to the battery and insert the bottom-meshed tin on top of the Prokopack aspirator.

6.4 Test the aspirator to confirm that it is working by ensuring that it is sucking in air.

6.5 The Prokopack aspirator is switched on and collection in done by systematically aspirating the walls and the areas under the roof using progressive down- and upward movements along the inside of each room in house

6.6 The collectors close the bottom- meshed tin and label appropriately (with collection ID date and time) before switching the aspirator off.

6.7 The collection tins containing the mosquitoes are placed in a bag and transported to the DVBNB laboratory.

**Text S4: Moran’s I Statistic**

We used the Global Moran’s I statistic to evaluate spatial autocorrelation of the total number of pupae observed in the households throughout the data collection period [6]. The Moran’s I allows us to evaluate the correlation of the pupae counts by comparing the counts of each household with counts in neighboring households. A significant result from the Moran’s I would suggest that not only are there specific households that produce more pupae than others, but also that these households cluster together in space, forming pupal productivity hotspots. Neighboring households were defined based on distance (distance-based neighbors) [7].

We used *150m* to define the distance band for a household’s neighbors (i.e. all households within 150m of the current household are defined as the current household’s neighbor) [8]. All derived neighbors were evenly weighted, as opposed to assigning farther neighbors smaller weights. Since the outcome is a count, the significance of the resulting statistic was evaluated using permutation tests [7]. In the permutation tests, the set of outcome values were randomly reassigned to the sample of households and a new Moran’s I is calculated. This was repeated 500 times. The random permutations of the outcome represent the distribution of the outcome under the null hypothesis. A p-value is derived by comparing the actual Moran’s I to the Monte-Carlo permutations at the *p = 0.05* level.

We tested the presence of spatial autocorrelation among each of the 20 houses within the 4 sites. Results are shown in Table S3. We also tested the sensitivity of the outcome to the distance threshold (*d = 50m – 500m,* Table S3). Finally, we evaluated if spatial autocorrelation varied by seasons. We did this by summing up the total pupae counts within a season for the households. These seasonal pupae count sums were then tested for spatial autocorrelation using the process described above. The results are shown in Table S4. The results suggest that for the houses sampled, pupae counts did not exhibit spatial correlation.

**References**

1. Guisan A, Harrell FE: **Ordinal response regression models in ecology.** *Journal of Vegetation Science* 2000, **11:**617-626.

2. Harrell FE: *Regression Modeling Strategies.* Springer-Verlag; 2006.

3. Wood S: *Generalized Additive Models An Introduction with R, Second Edition.* 2nd edn. Boca Raton, Florida: Chapman and Hall/CRC; 2017.

4. Umlauf N, Adler D, Kneib T, Lang S, Zeileis A: **Structured Additive Regression Models: An R Interface to BayesX.** *2015* 2015, **63:**46.

5. Brezger A, Kneib T, Lang S: **BayesX: Analyzing Bayesian Structural Additive Regression Models.** *2005* 2005, **14:**22.

6. Waller L, Gotway C: *Applied Spatial Statistics for Public Health Data.* Hoboken, New Jersey: John Wiley & Sons, Inc; 2004.

7. Bivand R, W. S. Wong D: **Comparing implementations of global and local indicators of spatial association.** *TEST* 2018, **27:**716 - 748.

8. Trpis M, Hausermann W: **Dispersal and other population parameters of *Aedes aegypti* in African village and their possible significance in epidemiology of vector-borne diseases** *American Journal of Tropical Medicine and Hygiene* 1986, **35:**1263-1279.


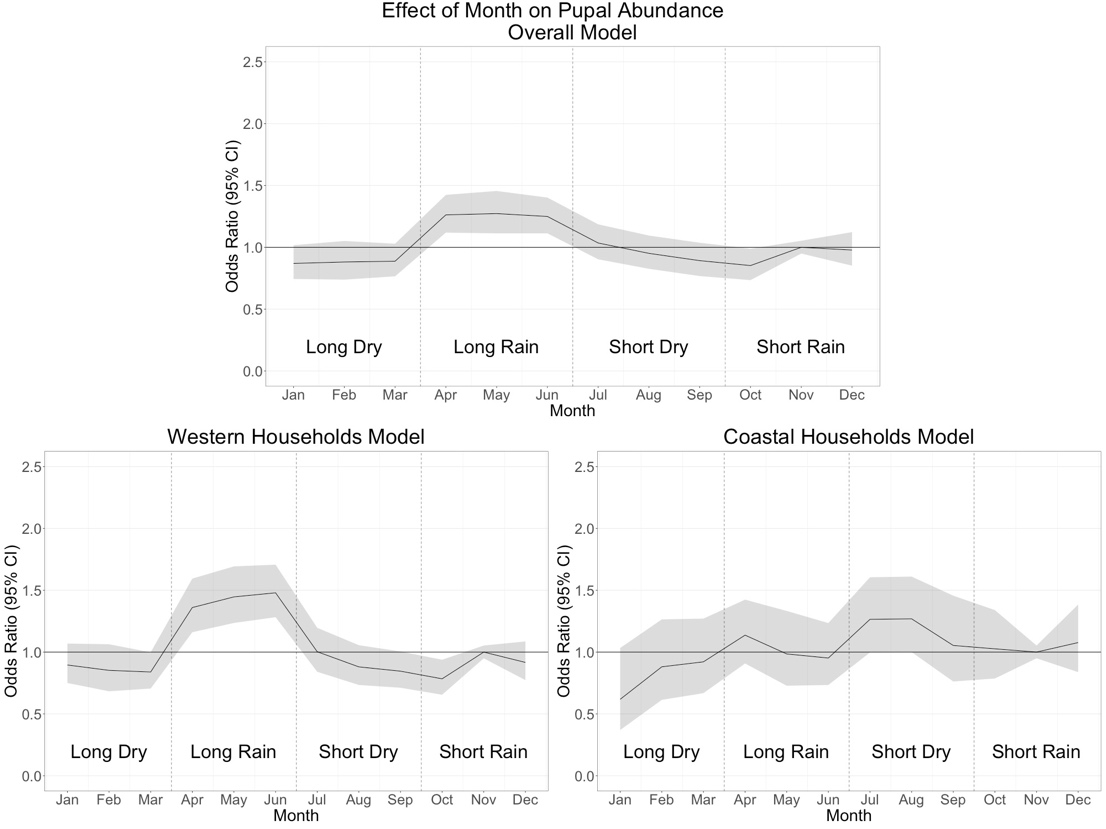


**Figure S1: Comparison of the seasonal effects in overall models with coastal and inland (western) models.**

Legend: (Top) Seasonal effect measured by month in the overall models. We observe strong seasonality, with risk of increasing abundance greatest in the long rainy season. (Right) Seasonal effect measured by month in the inland models. Similar to the overall models, risk of increasing abundance is greatest in the long rainy season. (Left) Seasonal effect measured by month in the coastal models. No strong seasonality is observed here. This suggests different seasonal patterns between the coast and the inland, with the inland exhibiting stronger seasonality.


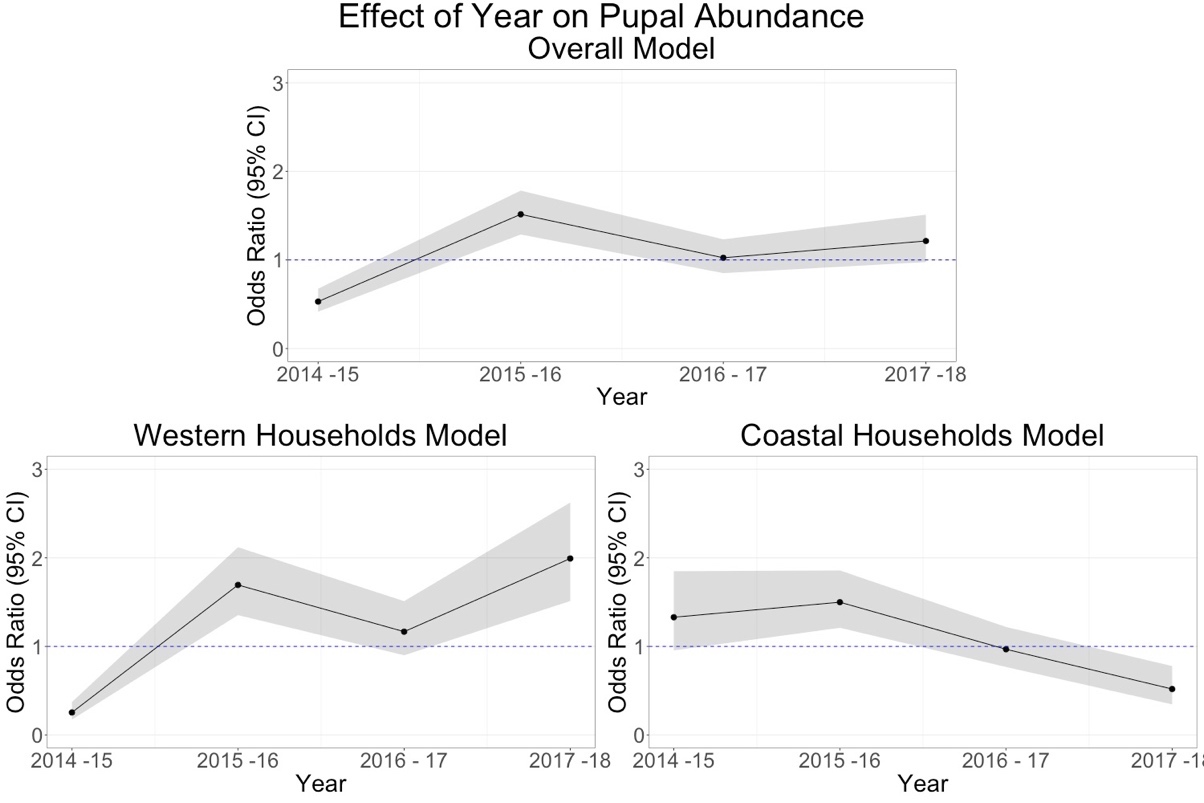


**Figure S2: Comparison of the effect of collection year in overall models with coastal and inland models.**

Legend: The figure shows the influence of year on pupal abundance in the overall model, and in the inland and coastal models. Year was included as a continuous term in the abundance models (i.e. year 1, 2, 3 and 4). The figure suggests some differences in influence of data collection year on abundance by location, with the inland model showing a general increase and the coast model showing a general decrease.

|  | **AIC** | **BIC** | **-Log-Lik** | **GCV** |
| --- | --- | --- | --- | --- |
| **Baseline (Month Only)** | 3264 | 3813 | 1543 | 0.80 |
| **Lag Term** | 3031 | 3578 | 1426 | 0.79 |
| **Month + Temp/Rain^a^** | 3246 | 3812 | 1532 | 0.79 |
| **Temp/Rain Only** | 3275 | 3781 | 1557 | 0.81 |
| **No Spatial term** | 3264 | 3817 | 1543 | 0.80 |
| **Temp/Rain + Month + Lag** | 3011 | 3579 | 1413 | 0.78 |

1. **Indicates the final model presented in the main analyses.**

**Table S1: Comparison of model fit between alternate models using AIC/BIC/GCV in the pupae abundance model.**

Legend: We ran separate models that included all the baseline demographic variables in addition to either month as the seasonal variable or temperature and rain as the seasonal variables. We also compared models that excluded the spatial term and models with a lag term. The lag term was not included in the final model because the authors reasoned that when a mixed-effects model framework is used for repeated measures data, any temporal correlation is captured by the random effect term (i.e. House ID). We compared the resulting models and chose the best model using model fit statistics.

| **Site** | ***d = 100m*** | | ***d = 150m*** | | ***d = 250m*** | | ***d = 500m*** | |
| --- | --- | --- | --- | --- | --- | --- | --- | --- |
|  | *Statistic* | *p-value* | *Statistic* | *p-value* | *Statistic* | *p-value* | *Statistic* | *p-value* |
| **Kisumu** | -0.01 | 0.48 | 0.00 | 0.40 | 0.00 | 0.43 | 0.02 | 0.18 |
| **Chulaimbo** | 0.02 | 0.38 | 0.07 | 0.16 | -0.18 | 0.83 | -0.08 | 0.59 |
| **Ukunda** | -0.04 | 0.61 | -0.10 | 0.68 | 0.10 | 0.17 | -0.06 | 0.49 |
| **Msambweni** | -0.05 | 0.77 | -0.13 | 0.86 | -0.14 | 0.80 | 0.08 | 0.12 |

**Table S2: Spatial Autocorrelation of Household Pupae Count using Moran’s I.**

Legend: We used the Moran’s I statistic to evaluate spatial autocorrelation of pupal abundance. Moran’s I checks if neighboring pairs within a distance *d* of one another, are more similar to one another in abundance than non-neighbor pairs (Appendix D). The range of Moran’s I statistics across different distance thresholds, suggests the absence of spatial autocorrelation.

| **Season** | **West** | | | | **Coast** | | | |
| --- | --- | --- | --- | --- | --- | --- | --- | --- |
|  | **Kisumu** | | **Chulaimbo** | | **Ukunda** | | **Msambweni** | |
|  | *Moran’s I* | *p-value* | *Moran’s I* | *p-value* | *Moran’s I* | *p-value* | *Moran’s I* | *p-value* |
| Cool, Dry | -0.03 | 0.47 | -0.03 | 0.50 | -0.06 | 0.67 | -0.09 | 0.66 |
| Long, Dry | -0.03 | 0.51 | 0.04 | 0.16 | 0.03 | 0.28 | -0.12 | 0.88 |
| Long, Rain | 0.02 | 0.35 | 0.09 | 0.11 | -0.04 | 0.53 | -0.02 | 0.52 |
| Short, Rain | -0.02 | 0.46 | 0.03 | 0.34 | -0.06 | 0.59 | -0.10 | 0.75 |

**Table S3: Evaluating within-seasonal spatial autocorrelation of household pupae count using Moran’s I for the 4 seasons.**

Legend: We used the Moran’s I with a distance threshold of 150m to evaluate within season spatial autocorrelation of pupal abundance in households for the 4 seasons. We found no evidence of spatial autocorrelation.

| **House**  **Characteristic** | **Full Model** | | **Western Households** | | **Coastal Households** | |
| --- | --- | --- | --- | --- | --- | --- |
|  | **OR** | **95% CI** | **OR** | **95% CI** | **OR** | **95% CI** |
| **Rooms** |  |  |  |  |  |  |
| **Less than 3** | *Ref* |  | *Ref* |  | *Ref* |  |
| **3 to 4** | 0.94 | [0.51, 1.71] | 0.34 | [0.08, 1.55] | 1.71 | [0.78, 3.75] |
| **4 or more** | 1.01 | [0.46, 2.25] | 3.19 | [0.24, 41.61] | 0.76 | [0.31, 1.89] |
| **No. of Sleepers** |  |  |  |  |  |  |
| **Less than 4** | *Ref* |  | *Ref* |  | *Ref* |  |
| **4 to 6** | 0.90 | [0.51, 1.60] | 0.84 | [0.19, 3.67] | 0.74 | [0.34, 1.64] |
| **7 or more** | 0.74 | [0.40, 1.36] | 0.75 | [0.09, 6.02] | 0.89 | [0.41, 1.96] |
| **House Wall** |  |  |  |  |  |  |
| **Mud** | *Ref* |  | *Ref* |  | *Ref* |  |
| **Cement** | 1.01 | [0.55, 1.85] | 0.96 | [0.13, 7.43] | 1.08 | [0.48, 2.43] |
| **House Roof** |  |  |  |  |  |  |
| **Iron Sheet** | *Ref* |  | *Ref* |  | *Ref* |  |
| **Grass** | 0.62 | [0.30, 1.25] | 0.22 | [0.00, 14.30] | 0.57 | [0.22, 1.47] |
| **Tile/Asbestos** | 1.19 | [0.42, 3.38] | 0.21 | [0.02, 1.76] | 1.06 | [0.12, 9.32] |
| **Room Ceilings** | 1.22 | [0.61, 2.44] | 0.68 | [0.16, 2.97] | 1.07 | [0.45, 2.53] |
| **Bushes/Tall Grass** | 1.60^*^ | [1.13, 2.28] | 1.54 | [0.86, 2.74] | 1.19 | [0.71, 2.00] |
| **Firewood Use** | 0.57^*^ | [0.37, 0.88] | 0.23. | [0.05, 1.09] | 0.67 | [0.40, 1.13] |
| **Eaves Open** | 2.57^**^ | [1.33, 4.95] | 2.61. | [0.89, 7.66] | 2.19 | [0.80, 6.02] |
| **Habitat Count** | 1.42^***^ | [1.21, 1.66] | 1.84^***^ | [1.41, 2.42] | 1.27 | [1.00, 1.60] |
| **Urban** | 1.67 | [0.31, 8.96] | 4.39 | [0.65, 29.58] | 0.87 | [0.25, 2.97] |
| **Insecticide/Coil^a^** | 0.76 | [0.32, 1.77] |  |  |  |  |
| **Location** | 0.88 | [0.15, 5.11] |  |  |  |  |

a) The Insecticide or Coil use variable was excluded from the location specific models due to small sample sizes.

* indicates p < 0.05, ** indicates p < 0.01, *** indicates p < 0.001

**Table S4: Model results. Comparison of risk factors for increased pupal abundance in coastal and western household models with complete model.**

Legend: In addition to the overall model of risk factors for pupal abundance we ran additional models with data from the coastal households and western households separately to evaluate any differences in the risk factors. The major risk factors for increased pupal abundance are relatively similar between the coastal and western models.

| **House**  **Characteristic** | **Original Model** | | **Household Replacements**  **Excluded** | | **Outliers**  **Excluded** | |
| --- | --- | --- | --- | --- | --- | --- |
|  | **OR** | **95% CI** | **OR** | **95% CI** | **OR** | **95% CI** |
| **Rooms** |  |  |  |  |  |  |
| **Less than 3** | *Ref* |  |  |  |  |  |
| **3 to 4** | 0.94 | [0.51, 1.71] | 0.64 | [0.25, 1.64] | 0.97 | [0.53, 1.78] |
| **4 or more** | 1.01 | [0.46, 2.25] | 0.95 | [0.27, 3.29] | 1.15 | [0.51, 2.59] |
| **No. of Sleepers** |  |  |  |  |  |  |
| **Less than 4** | *Ref* |  |  |  |  |  |
| **4 to 6** | 0.90 | [0.51, 1.60] | 1.14 | [0.55, 2.33] | 0.92 | [0.51, 1.64] |
| **7 or more** | 0.74 | [0.40, 1.36] | 1.24 | [0.52, 2.92] | 0.73 | 0.39, 1.36] |
| **House Wall** |  |  |  |  |  |  |
| **Mud** | *Ref* |  |  |  |  |  |
| **Cement** | 1.01 | [0.55, 1.85] | 1.31 | [0.43, 3.95] | 0.99 | [0.54, 1.84] |
| **House Roof** |  |  |  |  |  |  |
| **Iron Sheet** | *Ref* |  |  |  |  |  |
| **Grass** | 0.62 | [0.30, 1.25] | 0.57 | [0.14, 2.24] | 0.63 | [0.30, 1.30] |
| **Tile/Asbestos** | 1.19 | [0.42, 3.38] | 1.06 | [0.23, 4.80] | 1.12 | [0.39, 3.22] |
| **Room Ceilings** | 1.22 | [0.61, 2.44] | 0.96 | [0.40, 2.31] | 1.19 | [0.59, 2.41] |
| **Bushes/Tall Grass** | 1.60^*^ | [1.13, 2.28] | 2.28 | [1.49, 3.49] | 1.44 | [1.00, 2.07 |
| **Firewood Use** | 0.57^*^ | [0.37, 0.88] | 0.57 | [0.29, 1.09] | 0.55 | [0.35, 0.86] |
| **Eaves Open** | 2.57^**^ | [1.33, 4.95] | 2.42 | [1.06, 5.51] | 2.62 | [1.35, 5.08] |
| **Habitat Count** | 1.42^***^ | [1.21, 1.66] | 1.37 | [1.14, 1.65] | 1.45 | [1.23, 1.71] |
| **Insecticide/Coil^a^** | 0.76 | [0.32, 1.77] | 0.34 | [0.11, 1.12] | 0.73 | [0.31, 1.74] |
| **Location** | 0.88 | [0.15, 5.11] | 1.44 | [0.28, 7.44] | 0.96 | [0.14, 6.40] |
| **Urban** | 1.67 | [0.31, 8.96] | 3.20 | [0.78, 13.10] | 1.53 | [0.25, 9.47] |

* indicates p < 0.05, ** indicates p < 0.01, *** indicates p < 0.001

**Table S5: Sensitivity Analyses. Risk factors for pupal abundance and persistence after excluding household replacements or outliers (residual > 2) from overall model.**

Legend: We repeated the pupal abundance analysis excluding data from the replacement households. We also reran the model after excluding outliers, defined as observations generating residuals greater than 2. We observe some variation in the coefficients that are not statistically significant. The statistically significant coefficients do not show strong variation.
